# Identification of Major Signaling Pathways in Prion Disease Progression Using Network Analysis

**DOI:** 10.1371/journal.pone.0144389

**Published:** 2015-12-08

**Authors:** Khalique Newaz, K. Sriram, Debajyoti Bera

**Affiliations:** 1 Department of Computer Science, IIIT Delhi, New Delhi, India; 2 Center for Computational Biology, IIIT Delhi, New Delhi, India; Jaypee University of Information Technology, INDIA

## Abstract

Prion diseases are transmissible neurodegenerative diseases that arise due to conformational change of normal, cellular prion protein (PrP^*C*^) to protease-resistant isofrom (rPrP^*Sc*^). Deposition of misfolded Prp^*Sc*^ proteins leads to an alteration of many signaling pathways that includes immunological and apoptotic pathways. As a result, this culminates in the dysfunction and death of neuronal cells. Earlier works on transcriptomic studies have revealed some affected pathways, but it is not clear which is (are) the prime network pathway(s) that change during the disease progression and how these pathways are involved in crosstalks with each other from the time of incubation to clinical death. We perform network analysis on large-scale transcriptomic data of differentially expressed genes obtained from whole brain in six different mouse strain-prion strain combination models to determine the pathways involved in prion diseases, and to understand the role of crosstalks in disease propagation. We employ a notion of differential network centrality measures on protein interaction networks to identify the potential biological pathways involved. We also propose a crosstalk ranking method based on dynamic protein interaction networks to identify the core network elements involved in crosstalk with different pathways. We identify 148 DEGs (differentially expressed genes) potentially related to the prion disease progression. Functional association of the identified genes implicates a strong involvement of immunological pathways. We extract a bow-tie structure that is potentially dysregulated in prion disease. We also propose an ODE model for the bow-tie network. Predictions related to diseased condition suggests the downregulation of the core signaling elements (PI3Ks and AKTs) of the bow-tie network. In this work, we show using transcriptomic data that the neuronal dysfunction in prion disease is strongly related to the immunological pathways. We conclude that these immunological pathways occupy influential positions in the PFNs (protein functional networks) that are related to prion disease. Importantly, this functional network involvement is prevalent in all the five different mouse strain-prion strain combinations that we studied. We also conclude that the dysregulation of the core elements of the bow-tie structure, which belongs to PI3K-Akt signaling pathway, leads to dysregulation of the downstream components corresponding to other biological pathways.

## Introduction

Prion proteins are potential disease causing agents in a group of fatal neurodegenerative diseases which affects diverse group of species, including humans. Prions replicate by conversion of cellular prion protein (PrP^*C*^) to disease specific PrP^*Sc*^ isoforms. Accumulation of misfolded PrP^*Sc*^ proteins, rich in *β*-sheet structure, has been hypothesized to trigger a series of events which leads to neuronal dysfunction and death [1]. Many other neurodegenerative diseases such as Alzheimer’s or Parkinson’s diseases, are also due to the result of protein misfolding, and they are characterized by abnormal protein deposition and plaque formation [1, 2]. The pathological mechanisms related to prion disease may be relevant to other such neurological illness [3]. The similarities among these wide range of neurodegenerative diseases suggest that a thorough understanding of the molecular mechanisms related to prion diseases may prove to be of therapeutic importance to several other diseases.

Different strains of infectious prions, presumably arising from distinct structural forms of misfolded prion proteins, has different effects on disease progression (e.g. duration of incubation time, affected sites in the brain, etc.) [4]. This variation in the prion strains and their interactions with different host genotypes leads to variation in the pathogenesis of prion disease [4]. This poses a problem of identifying the core processes involved in the disease progression. To overcome this problem and to identify the shared biological modules that are affected by different prion-strain and host-genotype combinations, Hwang et al. [5] performed transcriptomic studies involving eight distinct mouse strain-prion strain models. They used five mouse strain-prion strain combinations and identified 333 differentially expressed core genes that showed high correlation in their patterns of differential expression throughout the disease progression. The core genes were further used to obtain dynamic protein interaction networks corresponding to different time-stamps in the disease progression. This mapping of differentially expressed genes to protein networks capture the temporal node dynamics of the network and also help in identification of the network modules being dysregulated as disease progresses.

Majority of the current methods [6] are dependent upon the static representation of protein interaction networks for analysis. This fails to capture the dynamic changes occurring within the cell. To investigate the changes with the prion disease progression, and also because different domain data can provide complementary biological insights [7], we integrate protein functional network data [8] with temporal gene expression data to computationally construct dynamic, time-specific protein networks [9]. Unlike Hwang’s work [5], in which network topology was not taken into consideration for the identification of the shared core genes, our work focus on utilizing the structure of time-stamped protein networks to identify the shared genes and the related biological pathways being involved in prion disease progression.

In this work, we address the question of core network-central pathways being involved in prion diseases, irrespective of the prion strain and host genotypes. We also look into the possibility of how these pathways may be involved in crosstalk during disease progression. We integrate time-specific differentially expressed genes corresponding to six mouse-prion models with static protein functional networks. These integrated networks show both node and edge dynamics in contrast to showing only node dynamics present in Hwang’s work. We use network theoretic concepts on these time-stamped networks to identify 148 shared DEGs common to five disease reproducing mouse-prion combinations. Relatively less number of shared DEGs in this work as compared to Hwang’s work is attributed to the procedural constraints applied to identify these genes. We consider only those DEGs as important whose corresponding proteins show considerable change in their topological properties in the disease related protein networks as compared to that in the protein networks related to a control mouse-prion model (mouse-prion combination which cannot develop the prion disease). Many signaling pathways and biological modules, including immunological pathway have been previously reported to be dysregulated during prion disease progression [10][11]. However, it is not clear which are the prime biological modules and pathways involved, and how these pathways interact and affect other pathways during prion disease pathogenesis. The shared 148 DEGs represent the genes whose related proteins are present at network-influential positions in the disease related protein networks, and hence the biological modules and pathways enriched in this set of DEGs should be considered important. Understanding the crosstalk between these pathways during disease progression can help in better understanding of the disease, and can also help in designing better therapeutic interventions [12]. We also identify a bow-tie structure being potentially affected during the disease condition. We propose an ODE model for this identified network structure which can be used as an abstract model to understand behavioral changes in the network components with different perturbations.

## Materials and Methods

**Fig 1:**
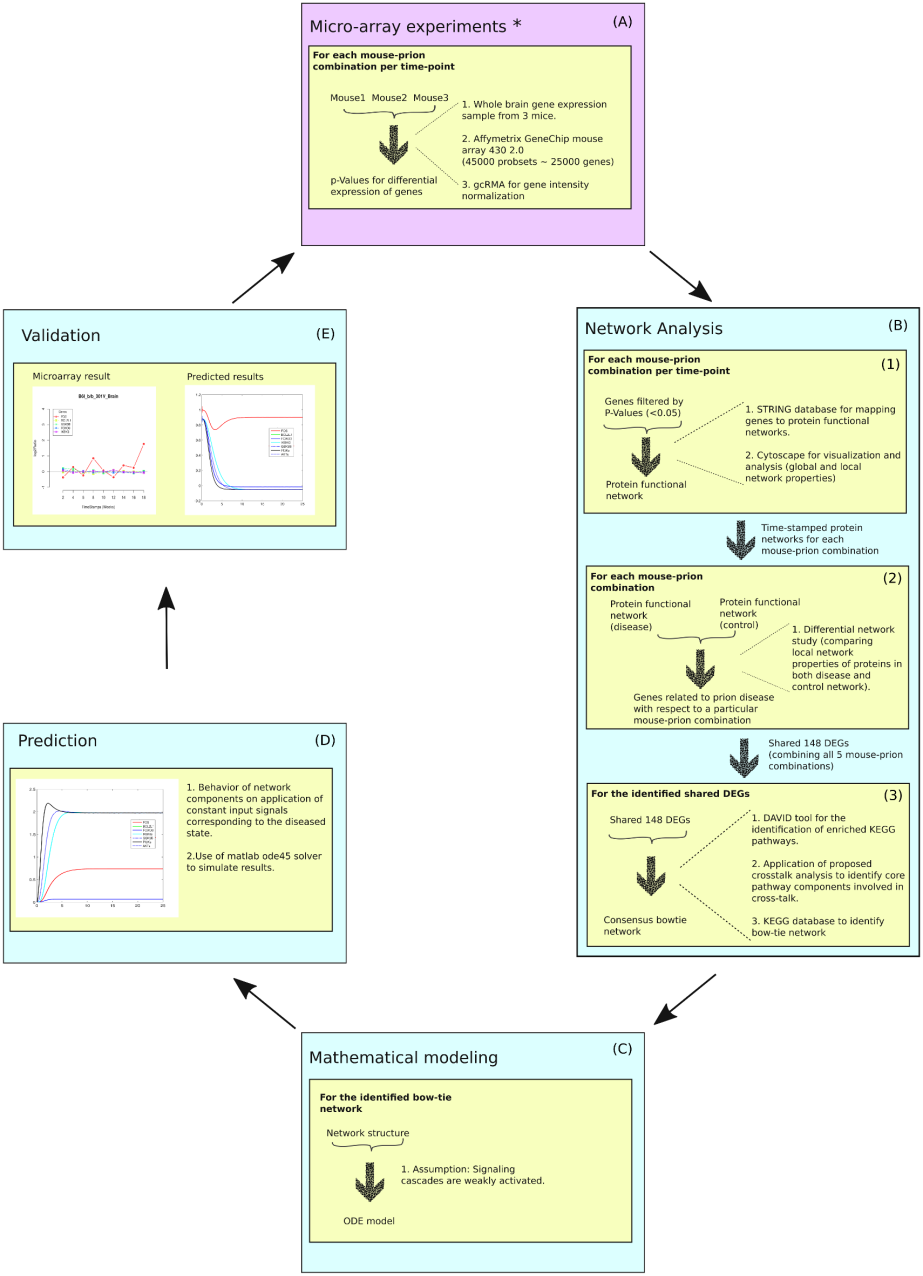
Basic workflow of this study. (A)We use microarray experiments to study differential gene expressions under specific disease conditions. (B.1)The DEGs are mapped to PPI networks using STRING database to get time-stamped PPI networks for every mouse-prion models. (B.2)Protein networks are used to identify potential disease related genes. (B.3)The identified shared DEGs are then used to identify genes potentially participating in crosstalk. These potential crosstalk genes are then mapped to KEGG database to identify a consensus bow-tie network. (C)Mathematical modeling of the identified bow-tie network using ordinary differential equations. (D) Prediction of the activities of the network components during disease condition. (E)Validation of predicted differential gene expression by comparing it with the microarray results. *The microarray experiments were performed in Hwang et al. [5].

Fig 1 outlines the basic methodology employed in this work. We start with microarray experiments to study differential expression of genes under a specific diseased condition from which we identify relevant biological networks. We then mathematically model the network to predict the behavior of the system under different conditions and validate the behavior with another set of microarray data obtained under similar conditions.

### Datasets

We obtain the gene expression data from the prion disease database (PDDB) [13]. This database contains all the information corresponding to the experiments performed by Hwang et al. [5]. PDDB also provides P-values for the differential expression of genes at different time-points corresponding to different mouse-prion models. In particular, we use 13,822 genes with their corresponding time-specific P-values in six different mouse-prion models. We obtain functional protein interaction networks corresponding to these time-specific DEGs using STRING database [8]. The STRING database maps a gene to a unique protein. We also use KEGG database [14] to retrieve information about the genes that belong to different biological pathways. Table 1 lists the details of six different mouse-prion models used in this work which represents a subset of the mouse-prion combinations used by Hwang et al. [5]. The microarray experiments performed by Hwang et al. [5] involved eight different mouse-prion combinations. Out of those eight, five combinations involved mouse having normal PrP expression, one with no PrP expression, and two with mutated (increased/decreased) levels of PrP expressions. Since the scope of this study is to highlight functionally relevant genes for prion disease, irrespective of the mouse genotype and prion strain, we include only those mouse-prion combinations for our study which involves mouse having normal PrP expression. It has been shown that the mouse with no PrP genotype cannot develop prion disease [15]. Hence, we use the mouse-prion model with mouse having no PrP genotype (FVB-0/0-RML), as the control combination to filter out DEGs not relevant for prion disease.

**Table 1:** Mouse strain-prion strain combinations^0^.

Mouse strain	Prnp genotype^1^	Prion strain	End point^2^	Harvest interval
C57BL/6J (BL6)	a/a	RML	23	2
C57BL/6J (BL6)	a/a	301V	41	4
C57BL/6I-1 (B6.I)	b/b	RML	48	4
C57BL/6I-1 (B6.I)	b/b	301V	18	2
FVB/Ncr (FVB)	a/a	RML	22	2
FVB.129-prnp (FVB)	0/0	RML	51	4

^0^The table lists different mouse-prion combinations used in this study.

^1^Host genotype for *PrP* ^*C*^ protein.

^2^Represents the clinical death of mouse.

### Protein functional networks (PFNs)

For every mouse strain-prion strain combination at a particular time-stamp, we identify DEGs by taking the genes having P-values less than the predefined threshold (< 0.05). We then map these time-specific DEGs to static protein functional interaction network using STRING database (version 9.1) to obtain time-specific protein networks that corresponds to a particular mouse-prion model. The edges in the functional protein networks obtained from the STRING database are weighted with a score from 100 to 1000 on the basis of their confidence, with 100 being least and 1000 being the highest confidence. The STRING database defines certain edge thresholds for retrieving networks of different confidence, that is, 150 for low confidence, 400 for medium confidence, 700 for high confidence, and 900 for highest confidence. To minimize false negative and false positive interactions in the retrieved protein functional association networks, we use a minimum edge confidence level of 700 to construct the networks. The networks we obtain mostly consists of a single large component and several small disconnected components. We use the largest connected component of the respective networks for further analysis. We provide the details about the number of nodes and interactions of the extracted networks (Tables C-H in S1 File).

### Global properties of time-stamped protein functional networks

We analyze the global properties of the time-specific protein functional networks using Cytoscape software [16]. In particular, we extract the information related to the following global properties, that is, average clustering coefficient, average shortest path length, centralization, density, and network heterogeneity.

### Network-influential disease genes

Local topological properties like degree, closeness centrality, and betweenness centrality can identify important nodes in biological networks [17, 18]. Comparing these local properties of the nodes in protein networks, corresponding to different biological conditions can help to identify proteins showing condition-specific network activity [19]. We compare time-specific protein networks corresponding to disease reproducing mouse-prion models with the protein networks of the control model. We use two approaches to identify network influential proteins pertaining to diseased condition. First, we identify the common proteins in the protein networks of diseased and the control state, and distinguish these proteins on the basis of their topological properties. Second, we identify unique proteins in the protein networks corresponding to the diseased condition.

We apply the notion of differential network centrality measures on the protein networks. We identify the common proteins showing high topological activity in the protein network of diseased combination as compared to the protein networks of the control combination. We compare local topological properties (degree, closeness centrality, betweenness centrality) of the common proteins in both the diseased and control protein networks. The aim is to identify proteins showing high network activity in disease related protein networks by measuring the centrality difference of the common proteins between control and disease related protein networks. We visualize the distribution of the centrality difference by plotting histograms of the calculated centrality difference against the number of genes. The plots show that the distribution is normal, with their mean close to zero (Figure C in S1 File). We choose to categorize the genes lying beyond the right standard deviation of the distribution as highly disease specific. Besides the common proteins, we also identify unique proteins showing high network centrality. We consider a unique protein as highly disease specific if it shows high degree centrality (having degree more than 10) in the disease related protein networks.

**Fig 2:**
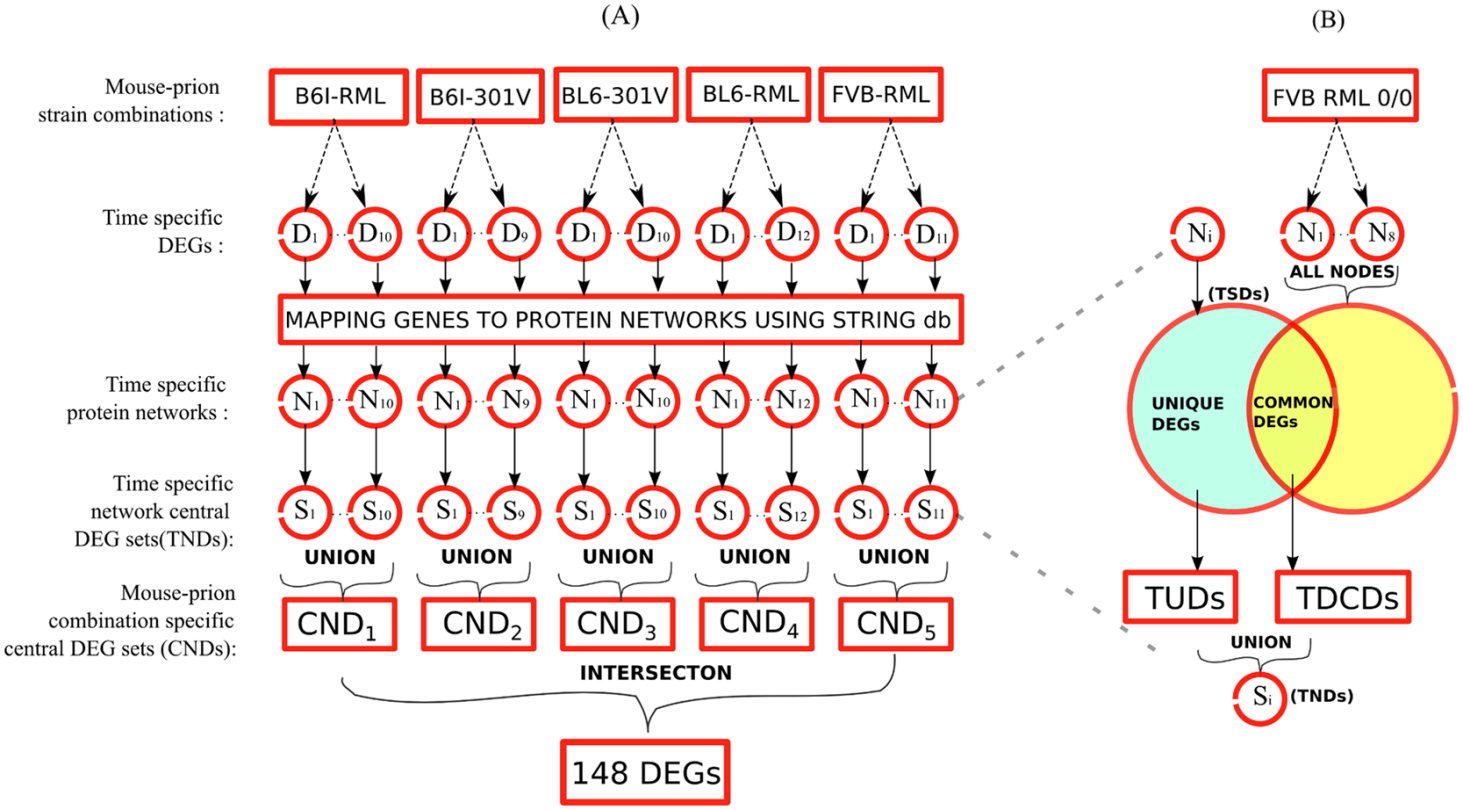
Outline of the work flow employed to identify network-central disease DEGs. Each DEG (differentially expressed gene) is mapped to unique protein using STRING database. (A) We obtain time-specific protein functional networks (N_*i*_) by mapping the timestamped DEGs (D_*i*_) to functional PPI networks from STRING database. We then combine network central DEGs (S_*i*_) for each time-specific networks to obtain network central DEGs corresponding to a particular mouse strain-prion strain combination (CND_*i*_). Finally, we combine all the CNDs to identify 148 DEGs which are common to atleast 4 mouse strain-prion strain combinations. (B) We compare each time-specific protein network (N_*i*_) corresponding to any of the 5 disease developing mouse-prion models with the network DEGs of the control combination. We identify two sets of DEGs. First set consists of the DEGs (TUDs) which are unique at a particular time-stamp and also present as hubs in the given DEGs network. Second set consists of the DEGs (TDCDs) which are common to both the diseased and control combinations but has relatively high centrality measures in diseased network compared to any of the time-specific networks corresponding to the control combination. Finally, we combine these two sets to give time specific network central DEGs (TNDs/S_*i*_) for each of the disease related protein networks.

Fig 2 outlines the procedure which we use to identify network influential proteins related to prion disease. we map time-specific DEGs (TSDs) to protein networks using STRING database. For a particular disease developing mouse-prion model, we compare its TSDs with all the DEGs (differentially expressed at any time-point) of the control combination (Fig 2B). We obtain time-specific differential common DEGs (TDCDs) by comparing topological properties of the corresponding proteins. These common DEGs corresponds to the common proteins present in the protein network of TSDs and all the protein networks of the control combination. We also identify unique DEGs among the TSDs to obtain time-specific unique DEGs (TUDs) (we only put a gene in TUDs set if its corresponding protein has a degree >10 in the protein network under consideration). We then combine both TUDs and TDCDs to get time-specific network influential DEGs (TNDs). For a particular mouse-prion model, we combine the corresponding TNDs to obtain combination specific network influential DEGs (CNDs) (Fig 2A). Finally, we combine all the five CNDs to identify 148 DEGs present in at least four of the CNDs sets. Algorithm 1 provides the pseudocode for obtaining TDCDs.

**Algorithm 1**: Selection of genes in TDCDs (Time-specific differential common DEGs) according to network centrality difference

1: **initialization**

2: N_*i*_: Network at time-stamp *i* corresponding to a particular mouse-prion model.

3: C_*i*_: Common DEGs between DEGs of N_*i*_ and all the DEGs of control combination.

4: M: Set of all time-stamped networks of the control combination.

5: Diff: Empty set for storing the centrality difference.

6: Nodes(): Function returning the set of nodes of the input network.

7: N_*i*_Map(): Maps the DEGs at time stamp i to their centrality values corresponding to the network at time stamp *i*.

8: S_*i*_: Empty set for storing DEGs selected as network-central for the time stamp *i*.

9: **Procedure** Centrality difference calculation

10:   **for** each DEG g in C_*i*_ **do**

11:    temp ← N_*i*_Map(g)   ▹ It can be betwenness, degree, or closeness centrality

12:    value ← 0

13:    **for** each network N_*j*_ in M **do**

14:     **if** *g* ∈ *Nodes*(*N*_*j*_) **then**

15:      value ← MAX(value, N_*j*_Map(g))

16:     **end if**

17:    **end for**

18:    Diff ← Diff ∪ (temp - value)         ▹ Storing the centrality differences

19:   **end for**

20:   Std ← standard deviation(Diff)   ▹ Calculating standard deviation of the data in Diff set

21:    Mea ← mean(Diff)         ▹ Calculating mean of the data in Diff set

22: **end procedure**

23: **Procedure** Selection of genes

24:   **for** each DEG g in C_*i*_ **do**

25:    temp ← N_*i*_Map(g)

26:    value ← 0

27:    **for** each network N_*j*_ in M **do**

28:     **if** g ∈ Nodes(N_*j*_) **then**

29:      value ← MAX(value, N_*j*_Map(g))

30:    **end if**

31:   **end for**

32:   temp ← temp - value

33:   **if** temp > (Std+Mea) **then**

34:    S_*i*_ ← S_*i*_∪ g   ▹ Storing genes in TDCDs for the network at time-stamp *i*

35:   **end if**

36:  **end for**

37: **end procedure**

We use degree, betweenness centrality, and closeness centrality as three different network centrality measures. Below, we define each of the three measures that we use and provide biological justification for their use.

Degree of a node is defined as the number of adjacent edges of that node in the network. The higher the degree of a node, the more central the node is in the corresponding network. Since our protein networks have “power-law” degree distributions (Figure B in S1 File), with many low-degree nodes and few high-degree nodes, and since removal of the high-degree nodes would impact the network structure (by disconnecting it), degree of a protein can be related to the protein’s essentiality as well as its involvement in disease [6, 20].

Closeness centrality of a node i in the network G is defined as $C_{i}^{c}=\frac{N-1}{\sum_{j\in G}d_{i,j}}$, where *d*_*i*,*j*_ is the shortest path between the nodes *i* and *j*, and *N* is the total number of nodes in graph *G*. It measures the “closeness” of a node to all other nodes in the network. According to the definition of the closeness centrality, the nodes with small shortest path distances to all other nodes have high network centrality. In a protein interaction network, closeness centrality of a protein indicates the “likelihood” of the protein to reach or be reachable from all other proteins [21]. And it is widely assumed that proteins that are closer to each other are more likely to perform similar functions [22].

Betweenness centrality of a node *i* in the network is defined as $C_{i}^{b}=\sum_{s\neq i\neq t}(\frac{\sigma_{st}\left(i\right)}{\sigma_{st}})$, where *s* and *t* are nodes from the network different from *i*, *σ*_*st*_ denotes the number of shortest path from *s* to *t*, and *σ*_*st*_(*i*) denotes the number of shortest path from *s* to *t* passing through *i*. It measures the involvement of a node in the shortest paths in the network. Intuitively, nodes that occur in many shortest paths have high centrality according to betweenness centrality. In a protein network, betweenness centrality of a protein indicates the “likelihood” of the protein to participate in pathways connecting all other proteins [23]. Removal of a protein that is on critical pathways between many other proteins could cause loss of communication between the proteins. Also, targeting such a node with a drug could cause the drug effects to spread fast to all the nodes [24].

### Pathways involved in the identified genes

We use the tool DAVID [25] to identify the enriched KEGG pathways for the identified 148 genes. DAVID tool employs a modified version of Fischer’s test to score the identified KEGG pathways in the set of input genes. We use P-value threshold of 0.05 for the modified Fischer’s test. Since affymetrix mouse array 430 2.0 chips were used for generating transcriptomic data in the works of Hwang et al. [5] and also since we use the same transcriptomic data for our network analysis, we use affymetrix mouse 430 2.0 genome as background for the purpose of KEGG pathway enrichment.

### Crosstalk candidate genes identification

We use the identified enriched pathways for further analysis to identify candidate genes potentially involved in crosstalk. We propose a procedure which exploits dynamic protein networks to identify crosstalk candidate genes. First, we identify all the genes belonging to the pathways under study (taking the genes involved in the identified pathways from the KEGG database and combining all the genes together to get a set of genes involved in at least one of the pathways). We intersect this set of genes with every time-stamped protein networks (corresponding to one of the disease reproducing mouse-prion models) to obtain dynamic protein networks related to the pathways under study. We then use extracted time-stamped protein networks to identify genes possibly involved in crosstalk.

To determine if a gene is involved in a crosstalk, we propose a method similar to the one used in Yang et al. [26] with a modification in the computation of crosstalk scores (CTSs). For every protein in a particular time-stamped protein network (the timed-stamped networks extracted as per the method explained in the previous paragraph), we calculate its relative crosstalk scores. Finally, we sum up the relative crosstalk scores of a particular protein corresponding to different time-stamps, and obtain its cross talk score. We calculate the relative score corresponding to every gene i at time-stamp t using the following expression.

$$\begin{array}{c} R_{i}^{t}=\frac{\sum_{e\in E(i)}M_{e}}{\left|E(i)|\cdot (n(n-1)\right)} \end{array}$$

Here, $R_{i}^{t}$ is the relative crosstalk score of protein *i* corresponding to the network at time-stamp *t*, *M*_*e*_ is the crosstalk score corresponding to the edge *e* which contains node *i*, *E*(*i*) is the set edges adjacent on node *i*, and *n* is the number of pathways under study. Both the nodes of an edge *e* can belong to more than one pathway, hence the edge can contribute for more than once in the relative crosstalk score of the genes associated with the corresponding nodes of the edge *e*. If *n* is the number of pathways under study, then highest score contributed by any edge to an adjacent node can be *n*(*n* − 1) (a node/protein can belong to all n pathways in consideration). Hence, the denominator for expression for calculating relative crosstalk scoring method comprises of |*E*(*i*)| ⋅ *n*(*n* − 1), which normalizes the score. Algorithm 2 provides the pseudocode for calculating crosstalk scores of every protein in the network.

**Algorithm 2**: Outline of the procedure used to identify the proteins possibly involved in the crosstalk.

1: **initialization**

2: P: Set of time-stamped protein networks corresponding to a particular mouse-prion model.

3: Path(g): Returns the set of pathways to which the gene g belongs.

4: Score_*p*_[g]: Stores the crosstalk score of gene g corresponding to a particular time-stamped protein network p. Initialized to zero for every gene.

5: Norm_*p*_[g]: Stores the normalization factor of gene g. Initialized to zero for every gene.

6: R[g]: Relative crosstalk score of gene g.

7: CTS[g]: Stores the crosstalk scores of the gene g. Initialized to 0 for every gene.

8: Paths: Set of all pathways used in the procedure to identify the possible crosstalk genes.

9: **procedure** Centrality difference calculation

10:  **for** each time-stamped network p in P **do**

11:   **for** every edge e in p **do** ▹ Loop for every edge in time-stamped network p

12:    g_1_ ← node 1 of edge e

13:    g_2_ ← node 2 of edge e

14:    **for** every pathway t in Paths **do**

15:     **for** every pathway k in Paths **do**

16:      **If** t != k **then**

17:       Norm_*p*_[g_1_] ← Norm_*p*_[g_1_] + 1 ▹ Storing the normalization value

18:       Norm_*p*_[g_2_] ← Norm_*p*_[g_2_] + 1

19:      **end if**

20:      **if** t != k AND t∈ Path(g_1_) AND k∈ Path(g_2_) **then**

21:       Score_*p*_[g_1_] ← Score_*p*_[g_1_] + 1

22:       Score_*p*_[g_2_] ← Score_*p*_[g_2_] + 1

23:      **end if**

24:     **end for**

25:    **end for**

26:   **end for**

27:   **for** every gene g in p **do**

28:    R[g] ← Score_*p*_[g]/(Norm_*p*_[g])     ▹ Relative crosstalk score calculation

29:    CTS[g] ← R[g] + CTS[g]         ▹ Storing crosstalk score

30:   **end for**

31:  **end for**

32: **end procedure**

**Fig 3:**
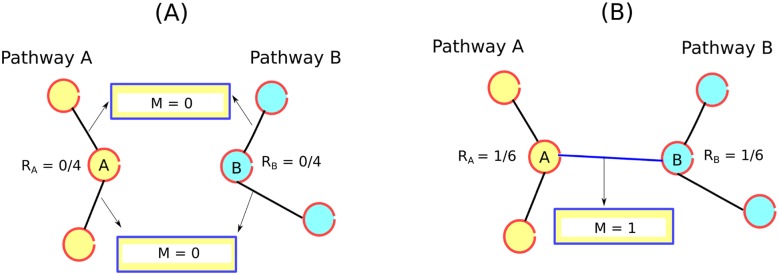
Relative crosstalk score. For a particular gene *i*, the relative crosstalk score is calculated as the ratio of inter-pathway edge’s scores and the normalizing factor (E(i) ⋅ n(n-1)). where, n is the number of pathways considered in the study, and E(i) is the number of adjacent edges of node i. In this example n is equal to 2. Hence, n(n-1) = 2. (A) Gene A and gene B are connected to the genes of their respective pathways. This results in relative crosstalk score for both gene A and gene B to be zero. (B) Gene A is connected to gene B present in different pathways. In this case, there is only one edge between pathway A and pathway B. The crosstalk score (M) corresponding to this edge, for both the genes is 1. Normalizing factor both the genes is E(i) ⋅ n(n-1) = 3 ⋅ 2 = 6. Hence the relative crosstalk score is 1/6 for both the gene A and gene B.

As an example for the calculation of the relative crosstalk scores at every time-stamp, consider a protein A in a hypothetical pathway A which is only connected to other proteins of the same pathway (Fig 3A). In this case, the relative crosstalk score of protein A will be 0. But if this protein A connects with a protein B of another hypothetical pathway B then the relative crosstalk scores of both the proteins A and protein B will have a non-zero value (Fig 3B). In general, if a protein has more functional associations with proteins of several other pathways in comparison to its own pathway, then it is considered a potential crosstalk candidate.

In this work, we perform the identification procedure for crosstalk candidate genes on two different mouse-prion models: B6.I-301V mouse-prion model, which has a short life span (18 weeks), and B6.I-RML model, which has a longer life span (48 weeks). We map some of the high crosstalk scoring genes to the protein signaling networks using KEGG database.

## Results and Discussion

### Global properties of networks

We expect that the effects of differential gene expressions in different mouse-prion models will reflect on the temporal protein functional networks. While the local topological properties (node centralities) show considerable change, we find negligible change in the global properties of the temporal networks corresponding to any of the mouse-prion models. We provide details of the global properties of the networks (Tables I-N in S1 File). Global properties including average clustering coefficients, and average degree of the time-specific protein networks do not change significantly with the disease progression. We also do not find significant change in the global properties of the disease related networks in comparison to the control networks (time specific protein functional networks related to FVB-RML-0/0 combination) (Figure A in S1 File). The degree distribution of most of the temporal networks follow power law with degree exponent close to 1.5 (Figure B in S1 File). Networks like these whose degree distribution is inversely proportional to the degree raised to some constant [27] (mathematically, *p*(*k*) ∝ *k* ^−*γ*^, where k is the degree of the node in the network and *γ* is the degree exponent) are called as scale-free networks. This scale-free property suggests that these networks are robust to random node failures [28].

### Network-influential shared DEGs via local topological properties

We identify 148 DEGs which shows high network activity in the protein functional networks of disease reproducing mouse-prion models. We find that many of these identified DEGs are involved in immunological response. As an infectious agent, PrP^*Sc*^ induces immune responses by activating innate immunity through glial cells (microglia and astrocytes) in the brain [29]. Microglia are among the earliest responders to any form of neurodegeneration [30]. Abnormal activation of microglia as a result of accumulation of aggregated molecules of PrP^*Sc*^ may lead to neuronal death [31]. Gómez-Nicola et al. [32] reports expansion of the resident microglial population during the pathological course of prion disease, which may contribute to disease progression. The activation of microglia results in the upregulation of proteins including complement factors, proteins of major histocompatibility complex, proinflammatory cytokines and interleukins [31]. Excessive and chronic activation of these factors produces oxidative stress, which can lead to neurotoxicity and subsequently to neurodegeneration. Activation of microglia also leads to activation of astrocytes [33, 34]. Role of astrocyte activation during prion neuroinflammation has been reported in Schultz et al. [35], in which astrocytes appear to be regulated by the interleukin-1 (IL-1) inducing astrocyte activation through CXCR3 ligand. The presence of genes related to the microglial and astrocytic activation (eg; *Cd68, Emr1*) in the shared 148 DEGs suggest that microglia not only proliferate in prion disease [36] but are also present as network-influential nodes in the disease related protein networks. Presence of glial markers such as *Gfap* and *P2ry* receptors in addition to *Csf1*, in the 148 shared DEGs indicates increment in the activities of astrocytes and leukocytes [37]. Other immune response related modules, that is, chemokines and cytokines also show high activity during prion disease progression. Detectable changes in the expression of CXC ligands even in the asymptotic stages of the disease suggest that the chemokines might play a pivotal role in promoting neurodegeneration in prion diseases [38].

**Fig 4:**
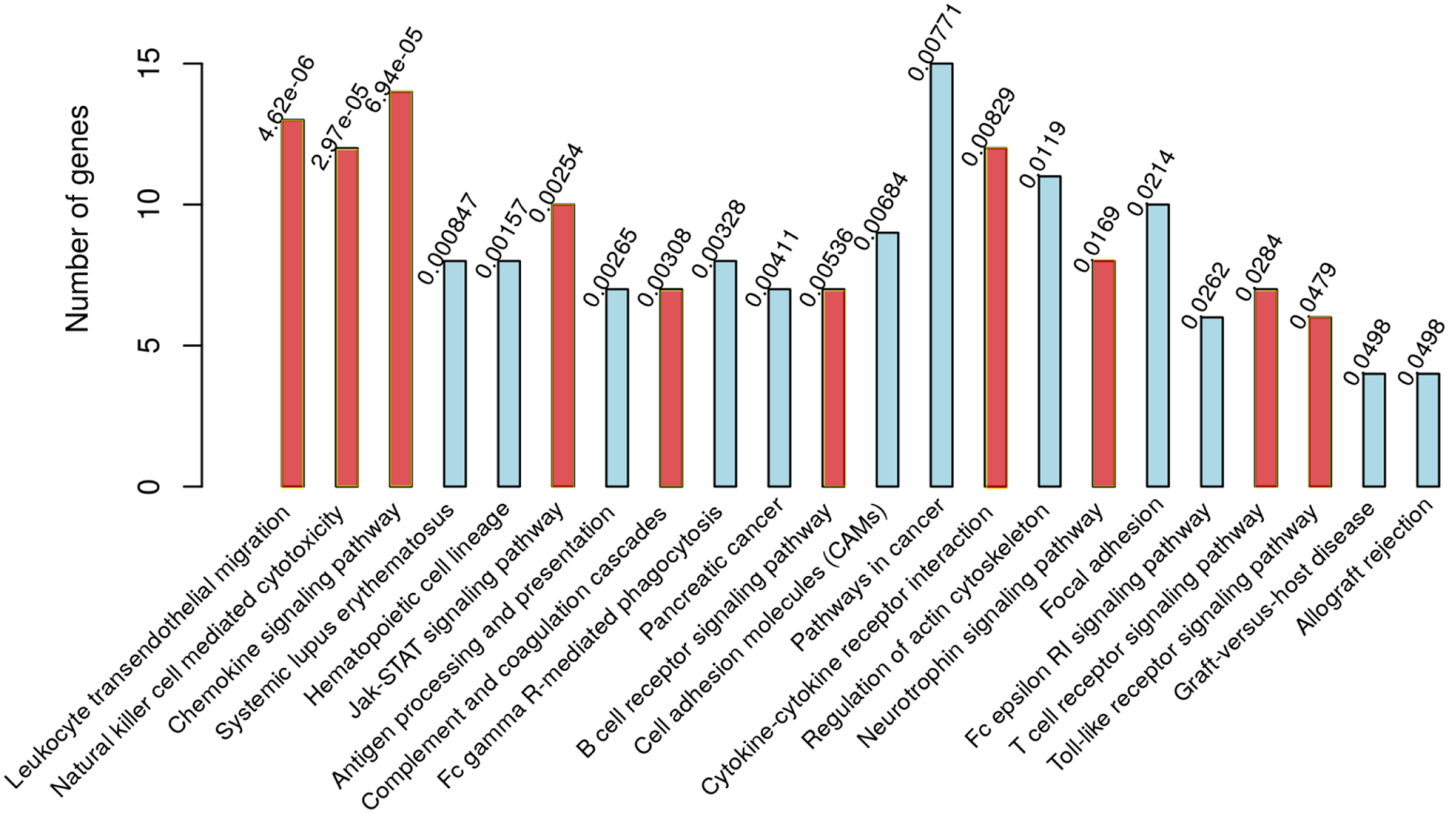
Network-central DEGs shows pathways involved in immunological response. Results of the KEGG pathway enrichment done on all 148 central DEGs identified. Red bars shows the pathways used for crosstalk analysis. The bottom of the bars show the KEGG pathway terms (P-values are reported on the top of the bars).

Our results include genes belonging to many of the biological modules related to immunological response including chemokines (eg; *Cxcl12, Cxcl16, Cx3cl1*), cytokines (eg; *Ccl9, Csf2ra, Csf1r*), neuroinflammation markers (*Gfap, Clec7a, Lgals3*), inflammatory cell types (*Cd44, Cd68, Ly86*) and genes that can be related to microglial activation (*Tyrobp, Lgals3, Osmr*) and astrocyte activation (*Gfap, Osmr*). This suggests that these biological modules are highly represented by the genes in the identified set 148 shared DEGs. Indeed, the pathway enrichment of these 148 shared DEGs highlights participation of pathways such as Leukocyte transendothelial migration, Cytokine-cytokine receptor interaction, Chemokine signaling pathway, and others, mainly related to immunological response (Fig 4). Table 2 shows some of the biological pathways represented by the shared 148 genes. Since, we identify these 148 genes by selecting network influential nodes of the corresponding protein networks, it suggests that the genes related to immunological response are present at network-influential positions in the protein networks related to prion disease progression. The presence of genes related to microglial and astrocytic activation along with the other genes related to immunological response, supports the hypothesis that neurotoxicity and neurodegeneration in prion disease results via excessive and chronic activation of microglia and other factors including complement factors, proteins of the major histocompatibility complex, pro-inflammatory cytokines and interleukins. We provide the list of 148 genes (Table A in S1 File). We also provide the details about mapping of the identified genes to biological pathways (Table B in S1 File). To visualize the interactions among the proteins corresponding to the 148 disease related shared genes, we integrate these 148 core genes with the protein functional networks. We then map the differential gene expression of B6.I-RML mouse-prion model to visualize the node dynamics with the disease progression (Figure D in S1 File). We observe that most of the genes in the disease network are highly upregulated towards the late stages of the disease.

**Table 2:** Some of the important biological pathways represented by the genes in shared 148 DEGs.

Biological pathway	Gene symbol	Description	Previous studies
Antigen processing and presentation	H2-D1	histocompatibility 2 D region	[39][40][5]
	H2-K1	histocompatibility 2 K1 K region	[39][5]
	H2-AB1	histocompatibility 2 class II antigen A beta 1	[5]
	H2-AA	histocompatibility 2 class II antigen A alpha	[5]
	Hspa4	heat shock protein 4	present work
	B2m	beta-2 microglobulin	[41][40][42][5]
	Cd74	CD74 antigen	present work
Natural killer cell mediated cytotoxicity	Fas	Fas (TNF receptor superfamily member 6)	present work
	Fcgr3	Fc receptor IgG low anity III	[39][5]
	Tyrobp	TYRO protein tyrosine kinase binding protein	[43][39][5]
	Lcp2	lymphocyte cytosolic protein 2	present work
	Pik3r1	phosphatidylinositol 3-kinase regulatory subunit polypeptide	present work
	Plcg2	phospholipase C gamma 2	present work
	Ptpn6	protein tyrosine phosphatase non-receptor type 6	[5]
	Vav1	vav 1 oncogene	present work
	Icam1	intercellular adhesion molecule 1	[39]
Leukocyte transendothelial migration	Actn1	actinin alpha 1	present work
	Ctnnd1	catenin (cadherin associated protein) delta 1	present work
	Gnai2	guanine nucleotide binding protein alpha inhibiting 2	present work
	Msn	moesin	[43][39][5]
	Ncf2	neutrophil cytosolic factor 2	present work
	Ncf4	neutrophil cytosolic factor 4	present work
Complement and coagulation cascades	A2m	alpha-2-macroglobulin	[43][39][5]
	C1qa	complement component 1 q subcomponent alpha polypeptide	[44][39][40][43][5]
	C1qb	complement component 1 q subcomponent beta polypeptide	[44][39][40][43][5]
	C1qc	complement component 1 q subcomponent C chain	[44][39][5]
	C3	complement component 3	[43][5]
	C3ar1	complement component 3a receptor 1	[44][39][43][5]
	Serping1	serine (or cysteine) peptidase inhibitor clade G member 1	[40][5]
Chemokine signaling pathway	Adcy7	adenylate cyclase 7	[5]
	Cxcl10	chemokine (C-X-C motif) ligand 10	[39][5]
	Cxcl12	chemokine (C-X-C motif) ligand 12	[39]
	Cxcl16	chemokine (C-X-C motif) ligand 16	[39]
	Cx3cl1	chemokine (C-X3-C motif) ligand 1	present work
	Stat1	signal transducer and activator of transcription 1	[5]
	Stat3	signal transducer and activator of transcription 3	[44][40][5]
Cytokine-cytokine receptor interaction	Ccl9	chemokine (C-C motif) ligand 9	[44][5]
	Csf1	colony stimulating factor 1 (macrophage)	[5]
	Csf1r	colony stimulating factor 1 receptor	[40][42][43][5]
	Csf2ra	colony stimulating factor 2 receptor alpha low-affinity	present work
	Kitl	kit ligand	present work
	Tgfb1	transforming growth factor beta 1	[41][5]
	Osmr	oncostatin M receptor	[44][43][5]
Neuroactive ligand-receptor interaction	Chrm1	cholinergic receptor muscarinic 1 CNS	present work
	Lpar1	lysophosphatidic acid receptor 1	present work
	Lpar6	purinergic receptor P2Y G-protein coupled 5	present work
	P2ry6	pyrimidinergic receptor P2Y G-protein coupled 6	[5]
	S1pr2	sphingosine-1-phosphate receptor 2	present work
	S1pr3	sphingosine-1-phosphate receptor 3	present work
	Trhr	thyrotropin releasing hormone receptor	present work
Neurotrophin signaling pathway	Rapgef1	Rap guanine nucleotide exchange factor (GEF) 1	present work
	Arhgdib	Rho GDP dissociation inhibitor (GDI) beta	[5]
	Gsk3b	glycogen synthase kinase 3 beta	present work
	Akt2	Protein kinase Akt-2	present work
	Trp53	transformation related protein 53	present work
	Crk	v-crk sarcoma virus CT10 oncogene homolog (avian)	present work
Glycerophospholipid metabolism	Dgka	diacylglycerol kinase alpha	present work
	Chka	choline kinase alpha	present work
	Pcyt1b	phosphate cytidylyltransferase 1 choline beta isoform	present work
	Chpt1	RIKEN cDNA 7120451J01 gene choline phosphotransferase 1	present work

Further, we compare our 148 shared DEGs with 333 shared DEGs identified in the work by Hwang et al. [5]. We find that there are many genes which belong to both the sets. The study performed by Hwang et al. tracked global gene expression in the brains of several mouse-prion models throughout the disease progression, and identified 333 shared common DEGs showing similar differential expression patterns over the life spans of five different mouse-prion models. In this work, we use the same five mouse-prion models to identify the shared DEGs involved in the prion disease progression. In contrast to the gene co-expression concept used in Hwang’s work, we use network theoretic approach to identify 148 shared DEGs. These 148 DEGs represent the genes which are potentially involved in the prion disease progression and show high network activity in the disease related protein functional networks. The central involvement of these genes in the disease related protein networks increases their potential disease involvement with respect to the ease of communication between them and the other network proteins. We find that out of 148 DEGs, 63 DEGs overlaps with the DEGs identified in the Hwang’s work. The overlapping 63 DEGs should be given more importance because of the fact that in addition to their behavior of high correlation in differential expression, they also show high network activity in the prion disease related protein functional networks. Identification of 85 DEGs which were not highlighted in the Hwang’s study reflects the fact that these genes, although did not show correlation in their differential expression patterns, are present at network-influential positions in protein networks. These genes should be considered important from point of view of the protein networks related to the disease.

### Crosstalk analysis of PFNs related to pathways

**Fig 5:**
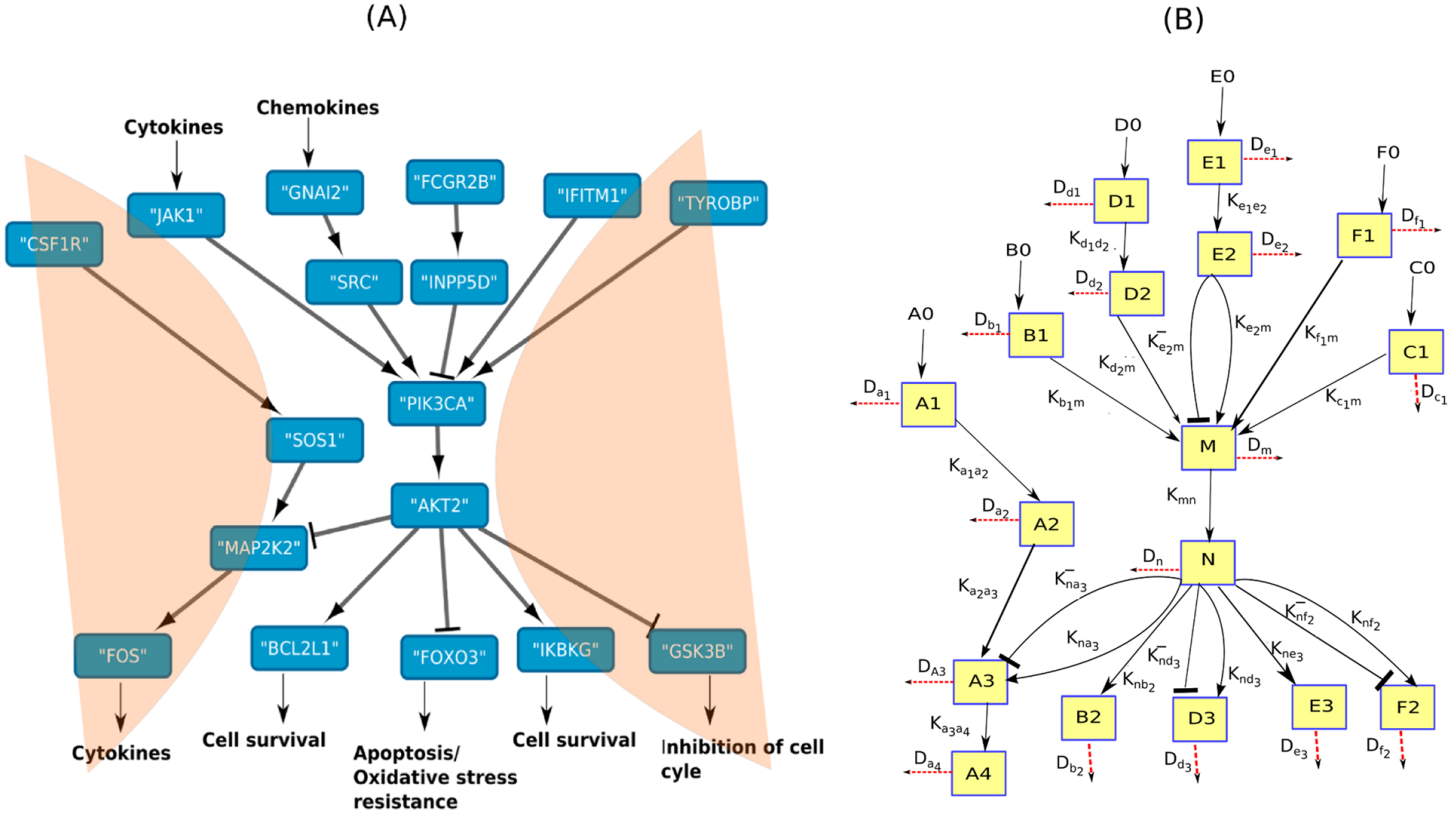
Identified bow-tie network and its schematic diagram. (A)The bow-tie structure identified from the protein functional networks. The pointed arrows represent the activation action by source onto the target gene and the flattened end arrows suggest inhibitory action. (B) Schematic representation of the structure used for ODE modeling. K_*d*_1_*d*_2__, K_*e*_1_*e*_2__, K_*a*_1_*a*_2__, K_*b*_1_*m*_, K_*d*_2_*m*_, K_*e*_2_*m*_, ${\text{K}}_{e_{2}m}^{-}$, K_*f*_1_*m*_ and K_*c*_1_*m*_ represents the upstream (input to the core of the bow-tie structure) rate constants of the signaling network. And K_*mn*_, K_*a*_2_*a*_3__, K_*na*_3__, ${\text{K}}_{na_{3}}^{-}$, K_*nb*_2__, K_*nd*_3__, ${\text{K}}_{nd_{3}}^{-}$, K_*ne*_3__, K_*nf*_2__ and ${\text{K}}_{nf_{2}}^{-}$ represents the downstream rate constants of the network structure. D_*a*1_, D_*a*_2__, D_*a*_3__, D_*a*_4__, D_*b*_1__, D_*b*_2__, D_*c*_1__, D_*d*_1__, D_*d*_2__, D_*d*_3__, D_*e*_1__, D_*e*_2__, D_*e*_3__, D_*f*_1__, D_*f*_2__, D_*m*_, and D_*n*_ represent the decay rate constant of the activated components A_1_, A_2_, A_3_, A_4_, B_1_, B_2_, C_1_, D_1_, D_2_, D_3_, E_1_, E_2_, E_3_, F_1_, F_2_, M, and N respectively. The constants K_*e*_2_*m*_, K_*nd*_3__, K_*na*_3__, K_*nf*_2__ represents the activation kinetic constants for basal production of the components M, D_3_, A_3_, and F_2_ respectively.

The pathways enriched in the DEGs identified in our study are mostly related to immunological response. To further understand the dynamic interactions between different signaling pathways in the obtained protein functional networks (PFNs), we calculate the CTSs (crosstalk scores) of every protein (see methods section). Proteins in this crosstalk analysis are grouped into 10 major identified pathways, that is, Chemokine signaling pathway, Toll-like receptor signaling pathway, Jak-STAT signaling pathway, Natural killer cell mediated cytotoxicity, T cell receptor signaling pathway, B cell receptor signaling pathway, Leukocyte transendothelial migration, Neurotrophin signaling pathway, Cytokine-cytokine receptor interaction, and Complement and coagulation cascades. We perform the identification procedure for crosstalk candidate genes on two different mouse-prion combinations: B6.I-301V mouse-prion model, which has a short life span (18 weeks), and B6.I-RML model, which has a longer life span (48 weeks). Table 3 lists the top 10 genes having high crosstalk scores corresponding to the combination B6.I-RML. Table 4 lists the top 10 genes having high crosstalk scores corresponding to the combination B6.I-301V. Also, the individual relative crosstalk scores of these genes are shown at every time-stamp of the prion disease progression. We observe that many of the high scoring crosstalk genes in both the combinations are common. This overlap implies that these genes, even though have been expressed via presumably different pathologies, are ranked as top scoring candidates in the crosstalk gene scoring criterion. These overlapping genes should be therefore, considered important. We map some of the high scoring genes to the signaling networks using KEGG database to obtain a bow-tie network (Fig 5A). The core elements in the identified bow-tie structure are the highly ranked genes according to the CTS scoring criterion.

**Table 3:** Top 10 high crosstalk scoring genes (B6.I-RML).

Gene	T1*	T2	T3	T4	T5	T6	T7	T8	T9	T10	CTSs^1^
PIK3CA	0.00	0.00	0.15	0.00	0.00	0.15	0.00	0.13	0.12	0.15	0.70
IFNAR2	0.00	0.00	0.05	0.09	0.03	0.09	0.08	0.09	0.08	0.11	0.62
IKBKG	0.17	0.00	0.14	0.00	0.07	0.06	0.10	0.00	0.00	0.04	0.58
VAV3	0.00	0.18	0.00	0.00	0.00	0.00	0.00	0.11	0.13	0.14	0.57
NFKBIA	0.11	0.14	0.15	0.00	0.00	0.00	0.09	0.05	0.00	0.00	0.55
AKT2	0.00	0.21	0.22	0.11	0.00	0.00	0.00	0.00	0.00	0.00	0.54
GSK3B	0.14	0.12	0.00	0.11	0.00	0.12	0.00	0.00	0.00	0.00	0.50
PIK3CD	0.19	0.00	0.00	0.00	0.11	0.19	0.00	0.00	0.00	0.00	0.48
PTPN11	0.00	0.10	0.00	0.09	0.00	0.12	0.00	0.00	0.00	0.13	0.44
PTPN6	0.00	0.00	0.00	0.00	0.00	0.00	0.10	0.07	0.08	0.09	0.34

*Ti represents the time-stamp i.

^1^CTSs represents the crosstalk scores of the genes.

**Table 4:** Top 10 high crosstalk scoring genes (B6.I-301V).

Gene	T1	T2	T3	T4	T5	T6	T7	T8	T9	CTSs
MAP2K2	0.31	0.00	0.00	0.00	0.39	0.00	0.00	0.00	0.00	0.70
AKT3	0.00	0.00	0.23	0.00	0.00	0.00	0.00	0.15	0.24	0.62
NFKBIA	0.00	0.00	0.11	0.11	0.10	0.06	0.06	0.00	0.11	0.56
IFNAR2	0.09	0.00	0.06	0.06	0.09	0.07	0.07	0.09	0.00	0.53
NFATC1	0.06	0.07	0.00	0.07	0.12	0.00	0.06	0.06	0.07	0.51
PIK3CD	0.15	0.00	0.18	0.00	0.18	0.00	0.00	0.00	0.00	0.51
GSK3B	0.11	0.16	0.00	0.09	0.10	0.00	0.00	0.00	0.00	0.46
SOS1	0.00	0.17	0.00	0.00	0.20	0.08	0.00	0.00	0.00	0.45
NFATC2	0.00	0.08	0.00	0.00	0.00	0.07	0.05	0.07	0.08	0.35
PIK3R1	0.00	0.15	0.18	0.00	0.00	0.00	0.00	0.00	0.00	0.33

Bow-tie structures are evolved, optimized architectures reflecting universal organizational principles of complex networks [45]. Several works have reported the existence of this universal structure at various biological levels, including metabolism [45] and immune system [46]. A nested bow-tie architecture of the molecular interactions in immune system is outlined in Kitano et al. [46], proposing that this structure helps in capturing wide range of molecular signatures and also helps in invoking appropriate counter measures. The robustness in biological systems are inherent property and it is necessary for the evolution of the system in changing environments [47]. The robustness of the bow-tie structure can be perceived by the fact that it facilitates control by accommodating various input perturbations and fluctuations on both spatial and temporal scales [45]. This heterogeneity in the inputs of the bow-tie structures allows for the robust regulation of the biological systems. In this work, we identify a bow-tie core signaling network which is prevalent in the prion disease progression. Since we perform crosstalk analysis on two different mouse-prion models to discover the bow-tie network, it may suggest that the prevalence of this structure in prion disease is a common phenomenon and is also independent of prion strain.

The core components (PI3Ks and AKTs) of the bow-tie network belong to the PI3K-Akt pathway. PI3K (phosphoinositide-3 kinase) has important functions in the immune system regulation. Receptors such as *Cd28* on T cells and *Cd19* on B cells activates the PI3Ks [48]. The products of PI3Ks, namely phosphatidylinositol3monophosphate (PIP3) and others govern many cellular events including cell growth and survival [49]. The activation of PI3K also downregulates the immune activity related to both innate and adaptive immune system [50], implying the crucial role of PI3ks in controlling the pro-inflammatory activity. The PI3K-Akt pathway regulates many other biological modules and pathways including Foxo signaling pathway [51]. Many other pathways affected directly or indirectly by the PI3K-Akt pathway have been reported to be dysregulated in prion disease. In Didonna et al. [52], the phosphorylation levels of *Src*, Mek 1/2 and Erk 1/2 signaling molecules, both before and after prion infection were assessed with the conclusion that prion replication leads to a hyperactivation of Erk1/2 pathway. In Simon et al. [53], the possibility of dysregulation of PI3K/AKT/GSK-3 pathway was explored. It was found that the prion strain altered PI3K-mediated signaling, evidenced by the results that the AKTs inhibition and *Gsk3b* activation were the common features of both in culture and in-vivo prion mediated neurotoxicity. We hypothesize that one of the consequences of prion disease is dysregulation of PI3Ks. Since PI3Ks comprises the core of the identified bow-tie structure, its dysregulation may affect other efferent pathways which can hinder in the proper regulation of important cellular functions including cell survival.

### Modeling the identified network

The genes involved in the immunological pathways identified in this study are part of different signaling cascades. These signaling pathways interact with each other via affecting other pathway’s output or by sharing common components. We identify a bow-tie network structure comprising of different signaling cascades having common components (PI3Ks and AKTs). The input components of this structure includes *Csf1r, Jak1, Gnai2, Fcgr2b, Ifitm1,* and *Tyrobp,* which belong to different signaling cascades. These independent cascades converge to a common crosstalk, that is, the core (PI3Ks and AKTs) of the bow-tie network. These core members are responsible for the transmission of input signals to the output components, that is, *Fos, Bcl2l1, Ikbkg, Foxo3,* and *Gsk3b*. In turn, these output components act as inputs to other regulatory functions including cell survival, apoptosis, oxidative stress resistance, and inhibition of cell cycle (Fig 5A). Hence, dysregulation of the functions of the core members of the bow-tie network may hinder in the proper functioning of these regulatory modules. Dysregulation of PI3K-Akt pathway in prion disease condition reflects the need to understand both its qualitative and quantitative behavior.

To understand the behavior of these interacting signaling cascades, we propose an ODE model given by the following equations. It is derived using the assumption that the signaling cascades are weakly activated [54, 55]. We use Matlab ode45 solver for model simulation.

$$\begin{array}{c} \frac{d[A_{1}]}{dt}=\left[A_{0}\right]-D_{a_{1}}\cdot \left[A_{1}\right] \end{array}$$ (1)

$$\begin{array}{c} \frac{d[A_{2}]}{dt}=K_{a_{1}a_{2}}\cdot \left[A_{1}\right]-D_{a_{2}}\cdot \left[A_{2}\right] \end{array}$$ (2)

$$\begin{array}{c} \frac{d[A_{3}]}{dt}=K_{a_{2}a_{3}}\cdot \left[A_{2}\right]-K_{na_{3}}^{-}\cdot \left[N\right]\cdot \left[A_{3}\right]+K_{na_{3}}\cdot \left[N\right]-D_{a_{3}}\cdot \left[A_{3}\right] \end{array}$$ (3)

$$\begin{array}{c} \frac{d[A_{4}]}{dt}=K_{a_{3}a_{4}}\cdot \left[A_{3}\right]-D_{a_{4}}\cdot \left[A_{4}\right] \end{array}$$ (4)

$$\begin{array}{c} \frac{d[B_{1}]}{dt}=\left[B_{0}\right]-D_{b_{1}}\cdot \left[B_{1}\right] \end{array}$$ (5)

$$\begin{array}{c} \frac{d[B_{2}]}{dt}=K_{nb_{2}}\cdot \left[N\right]-D_{b_{2}}\cdot \left[B_{2}\right] \end{array}$$ (6)

$$\begin{array}{c} \frac{d[C_{1}]}{dt}=\left[C_{0}\right]-D_{c_{1}}\cdot \left[C_{1}\right] \end{array}$$ (7)

$$\begin{array}{c} \frac{d[D_{1}]}{dt}=\left[D_{0}\right]-D_{d_{1}}\cdot \left[D_{1}\right] \end{array}$$ (8)

$$\begin{array}{c} \frac{d[D_{2}]}{dt}=K_{d_{1}d_{2}}\cdot \left[D_{1}\right]-D_{d_{2}}\cdot \left[D_{2}\right] \end{array}$$ (9)

$$\begin{array}{c} \frac{d[D_{3}]}{dt}=K_{nd_{3}}\cdot \left[N\right]-K_{nd_{3}}^{-}\cdot \left[N\right]\cdot \left[D3\right]-D_{d_{3}}\cdot \left[D_{3}\right] \end{array}$$ (10)

$$\begin{array}{c} \frac{d[E_{1}]}{dt}=\left[E_{0}\right]-D_{e_{1}}\cdot \left[E_{1}\right] \end{array}$$ (11)

$$\begin{array}{c} \frac{d[E_{2}]}{dt}=K_{e_{1}e_{2}}\cdot \left[E_{1}\right]-D_{e_{2}}\cdot \left[E_{2}\right] \end{array}$$ (12)

$$\begin{array}{c} \frac{d[E_{3}]}{dt}=K_{ne_{3}}\cdot \left[N\right]-D_{e_{3}}\cdot \left[E_{3}\right] \end{array}$$ (13)

$$\begin{array}{c} \frac{d[F_{1}]}{dt}=\left[F_{0}\right]-D_{f_{1}}\cdot \left[F_{1}\right] \end{array}$$ (14)

$$\begin{array}{c} \frac{d[F_{2}]}{dt}=K_{nf_{2}}\cdot \left[N\right]-K_{nf_{2}}^{-}\cdot \left[N\right]\cdot \left[F2\right]-D_{f_{2}}\cdot \left[F_{2}\right] \end{array}$$ (15)

$$\begin{array}{c} \frac{d[N]}{dt}=K_{mn}\cdot \left[M\right]-D_{n}\cdot \left[N\right] \end{array}$$ (16)

$$\begin{array}{ccc} \frac{d[M]}{dt} & = & K_{b_{1}m}\cdot \left[B_{1}\right]+K_{c_{1}m}\cdot \left[C_{1}\right]+K_{d_{2}m}\cdot \left[D_{2}\right]\\ & & -K_{e_{2}m}^{-}\cdot \left[E_{2}\right]\cdot \left[M\right]+K_{e_{2}m}\cdot \left[E_{2}\right]+K_{f_{1}m}\cdot \left[F_{1}\right]-D_{m}\cdot \left[M\right] \end{array}$$ (17)

The schematic diagram of the network is outlined in Fig 5B. The network comprises of six different signaling cascades with two common components (M and N). Activation of component M activates N and as a result, N leads to activation/deactivation of other downstream components. K_*d*_1_*d*_2__, K_*e*_1_*e*_2__, K_*a*_1_*a*_2__, K_*b*_1_*m*_, K_*d*_2_*m*_, K_*e*_2_*m*_, ${\text{K}}_{e_{2}m}^{-}$, K_*f*_1_*m*_ and K_*c*_1_*m*_ represents the upstream (input to the core of the bow-tie structure) rate constants of the signaling network. And K_*mn*_, K_*a*_2_*a*_3__, K_*na*_3__, ${\text{K}}_{na_{3}}^{-}$, K_*nb*_2__, K_*nd*_3__, ${\text{K}}_{nd_{3}}^{-}$, K_*ne*_3__, K_*nf*_2__ and ${\text{K}}_{nf_{2}}^{-}$ represents the downstream rate constants of the network structure. D_*a*1_, D_*a*_2__, D_*a*_3__, D_*a*_4__, D_*b*_1__, D_*b*_2__, D_*c*_1__, D_*d*_1__, D_*d*_2__, D_*d*_3__, D_*e*_1__, D_*e*_2__, D_*e*_3__, D_*f*_1__, D_*f*_2__, D_*m*_, and D_*n*_ represent the decay rate constant of the activated components A_1_, A_2_, A_3_, A_4_, B_1_, B_2_, C_1_, D_1_, D_2_, D_3_, E_1_, E_2_, E_3_, F_1_, F_2_, M, and N respectively. The constants K_*e*_2_*m*_, K_*nd*_3__, K_*na*_3__, K_*nf*_2__ represents the activation kinetic constants for basal production of the components M, D_3_, A_3_, and F_2_ respectively. The model provides an opportunity to study the behavior of these interacting pathways which are potentially affected during prion disease progression.

**Fig 6:**
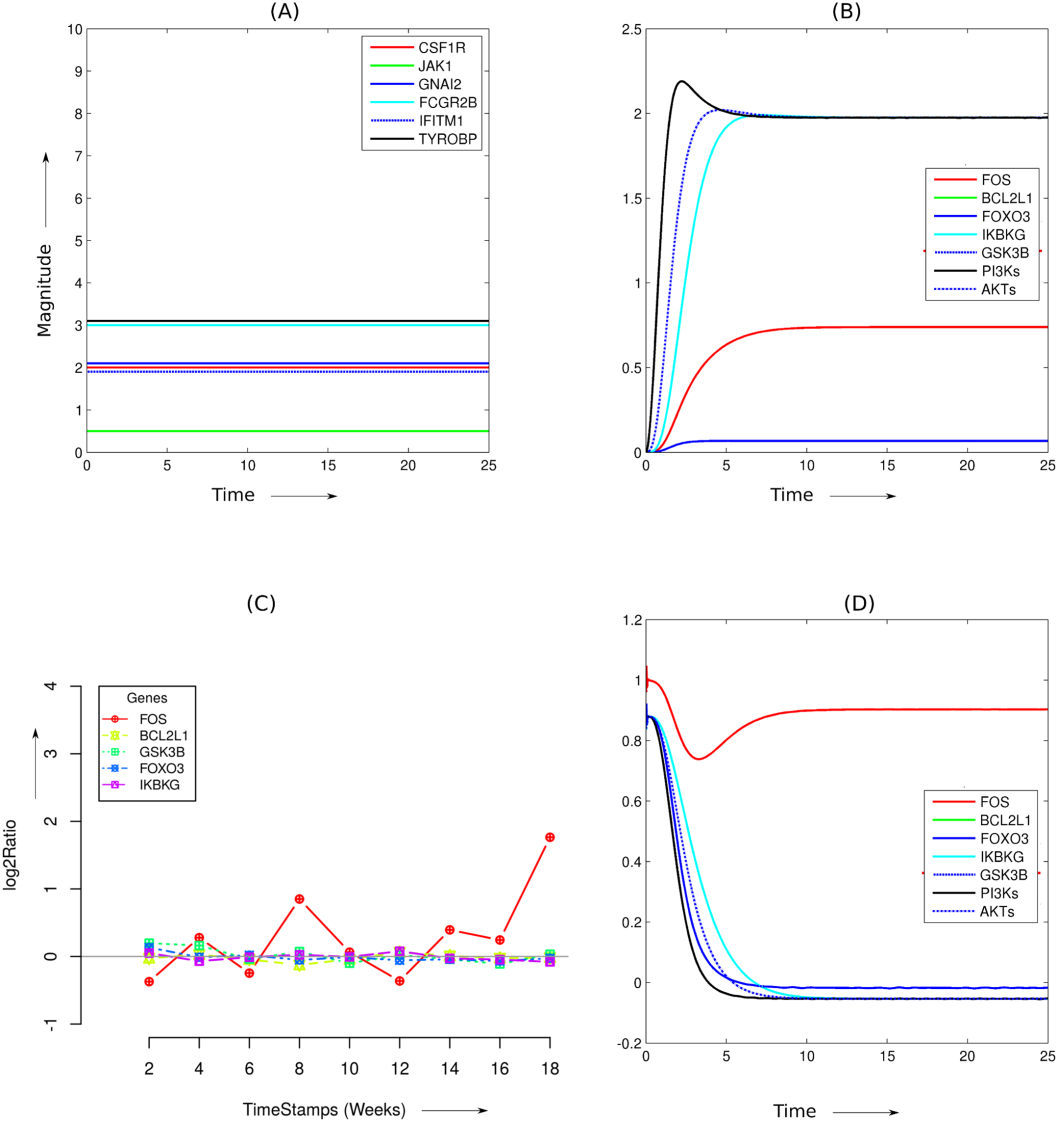
Comparison of microarray results with the numerical simulations of the proposed model. (A) We use constant input signals to observe the behavior of some of the network components of the identified bow-tie signaling network structure. The magnitude of these signals corresponds to the amount of signals of network components *Tyrobp, Gnai2, Ifitm1, Jak1, Fcgr2b,* and *Csf1r,* as shown by their differential expressions in the works of Hwang et al. [5]. For most of the mouse-prion combination models, the differential expression pattern of these input gene components are approximately similar. (B) The output signals show the behavior of some of the network components when we apply constant input signals corresponding to the diseased state. (C) Differential expression pattern of the output network components corresponding to the mouse-prion model B6I-301V. This result is taken from the microarray results of the work carried out in Hwang et al. Plots of differential expression of these genes corresponding to other mouse-prion models is given in Figure E in S1 File. (D) Model predictions of the differential expression pattern of the same network components (*Fos, Bcl2l1, Ikbkg, Foxo3, and Gsk3b*).

In the identified bow-tie network, the input components, that is, *Csf1r, Jak1, Gnai2, Fcgr2b, Ifitm1,* and *Tyrobp* corresponds to A_1_, B_1_, D_1_, E_1_, F_1_, and C_1_ respectively in the schematic diagram (Fig 5). We apply constant input signals (Fig 6A), whose magnitude corresponds to the amount of gene expressions (as shown by the microarray results corresponding to the works of Hwang et al.) at later stages of the disease. For most of the genes corresponding to the input network components, their differential expression pattern across all five disease reproducing mouse-prion models are similar. Fig 6B shows simulation results of various outputs signals in response to the applied constant input signals (corresponding to the diseased condition). Fig 6C shows microarray results of the differential expression pattern of these output network components corresponding to B6.I-301V mouse-prion combination. As evident form the gene expression profiles, *Fos* is relatively up-regulated in comparison to other genes related to output components of the modeled network. Other genes, including *Bcl2l1*, *Gsk3b*, *Foxo3*, and *Ikbkg* does not show considerable differential gene expression during any stage of the disease progression. Fig 6D shows the model predictions for differential expression of the output components. We capture the differential expression via log-2-ratio of the outputs corresponding to the diseased and the normal condition. The simulation results also indicate up-regulation of *Fos* and relatively down-regulation of other components of the modeled network. Both *Bcl2l1* and *Ikbkg*, which have been shown to take part in cell survival [56], show negligible differential expression in both the simulation and microarray results. Likewise, *Foxo3* and *Gsk3b* show no change in the diseased condition, as evident from their differential expression results of both microarray and numerical simulations. Also the core components of the identified bow-tie network, that is, *PI3Ks* and *AKTs* show negligible differential expression in the diseased state (Fig 6D). Here, we propose a phenomenological model which captures the effects of different input combinations. We assign arbitrary values to the rate constants. The model can be improved based on new experiments related to prion disease progression.

## Conclusion

Prion disease, a neurodegenerative disorder, is caused by the structural change in the cellular prion protein that leads to dysregulation of many biological pathways, and ultimately resulting in neuronal death. Different strains of PrP^*Sc*^ interacts differently with different hosts depending upon host’s genotype which leads to variation in the disease pathologies. Irrespective of these variations, the clinical symptoms for the disease are same, indicating the involvement of common biological modules and pathways. Identification of these core biological modules and pathways that are dysregulated in the prion disease will prove to be important in understanding other neurodegenerative diseases that show similar behavior.

In this study we integrate both protein functional interaction networks and gene expression profiles related to five different mouse-prion models to obtain dynamic protein interaction networks and perform network analysis to gain insights about the disease progression. We use local topological properties of the nodes in the dynamic protein networks to identify 148 genes related to the prion disease. The proteins corresponding to these 148 shared genes show high network centrality behavior in the disease related protein networks in comparison to the protein networks related to control combination (prion-host genotype combination which cannot develop prion disease) and should be considered important from the point of view of the disease related protein networks. Enrichment of immunological pathways in the shared genes highlights that the pathways related to immunological response are not only activated in the prion disease progression, but they also occupy network-influential positions in the disease related protein networks at later stages of the disease progression.

We also propose a crosstalk gene ranking method which utilizes dynamic protein networks to identify potential genes involved in possible crosstalks. We use the genes related to the identified immunological pathways to construct dynamic protein networks and identify several high scoring genes as possible crosstalk candidates. We use KEGG database to map some of the high crosstalk scoring genes on to the biological pathways and identify a bow-tie signaling network structure potentially dysregulated in prion disease. The bow-tie architectures provide a homeostatic environment by channeling the heterogeneous inputs through its core elements, which integrates the fluctuations in the input and provides a controlled output. The core of the bow-tie structure identified in this work consists of genes related to PI3K-Akt signaling pathway. Since PI3K-Akt signaling pathway regulates many crucial biological functions in the cell [49], we propose that its dysregulation in the prion disease is one of the major consequences of the disease. We also propose a mathematical model for the identified bow-tie signaling network. Initial simulations of the proposed ODE model suggest downregulation of the genes involved in crucial biological functions including apoptosis and cell survival. The model is based on mass action kinetics which can be used as an abstract model to study the effects of different perturbation in the network. In future, we intend to further refine the model and validate it against other experiments for better predictions.

## Supporting Information

S1 File This file contains Figures A-E, and Tables A-N. (PDF)

S1 Result B6I-RML related DEGs with cross-talk scores. This file contains the ranked list (according to the cross-talk score) of all the genes expressed at any time-point of the disease progression in the mouse-prion model B6I-RML.
(CSV)

S2 Result B6I-301V related DEGs with cross-talk scores. This file contains the ranked list (according to the cross-talk score) of all the genes expressed at any time-point of the disease progression in the mouse-prion model B6I-301V.
(CSV)

S1 Dataset List of 13,822 genes used in this work. The differential expression of these genes were tracked in every mouse-prion model and used for all the experiments in this work.
(CSV)

S2 Dataset Files containing probeset ids and their corresponding P-values. This file contains the text files corresponding to every six mouse-prion model used in this work. These files contain the probeset ids of affymetrix mouse 430 2.0 array, and the corresponding P-values for their differential expression at every sampled time-point.
(ZIP)
