# Supplementary material for: Identification of Major Signaling Pathways in Prion Disease Progression Using Network Analysis
Source: PLoS One. 2015 Dec 8;10(12):e0144389. doi: 10.1371/journal.pone.0144389 (PMC4672924; doi:10.1371/journal.pone.0144389)
Supplement: S1 File — (PDF) [file pone.0144389.s001.pdf]

# Supplementary Information

## List of Contents

Figure A: Temporal change in the global properties (average degree and average clustering coefficient) of the protein networks corresponding to six different mouse-prion models.

Figure B: Degree distributions of the temporal networks corresponding to the mouse-prion model B6.I_b/b_RML_Brain.

Figure C: Histogram corresponding to centrality difference.

Figure D: Network of 148 shared genes showing the node dynamics at initial and final stages of the disease.

Figure E: Comparison of microarray results and model predictions for the output components of the identified bow-tie signaling network structure.

Table 1: List of 148 genes identified in this study

Table 2: List of pathways which can be associated with the identified set of 148 genes.

Table 3-9: Network structure information for different mouse strain-prion strain combinations.

Table 10-14: Global properties details for different mouse strain-prion strain combinations.

**Supplementary figures:**

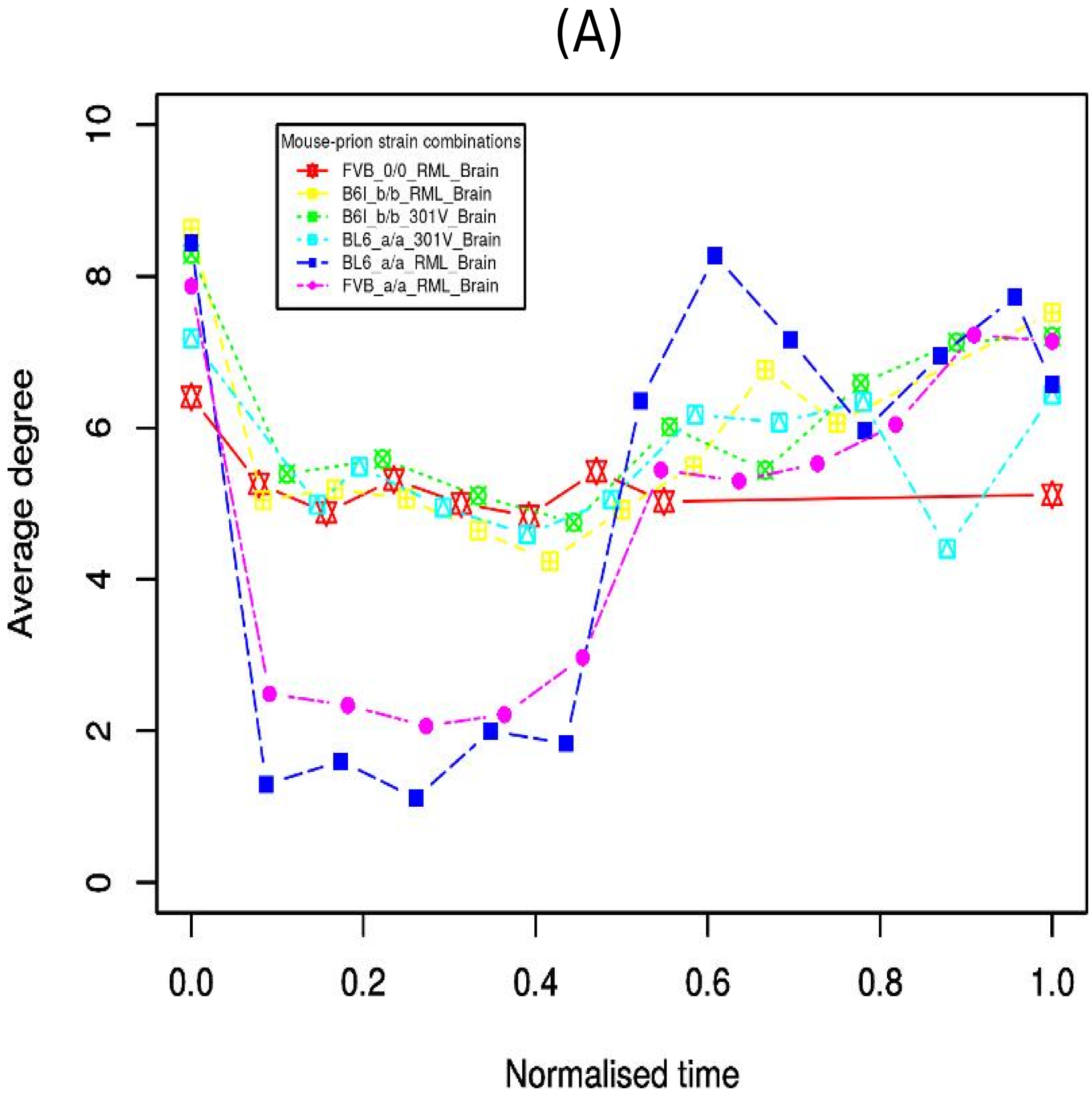

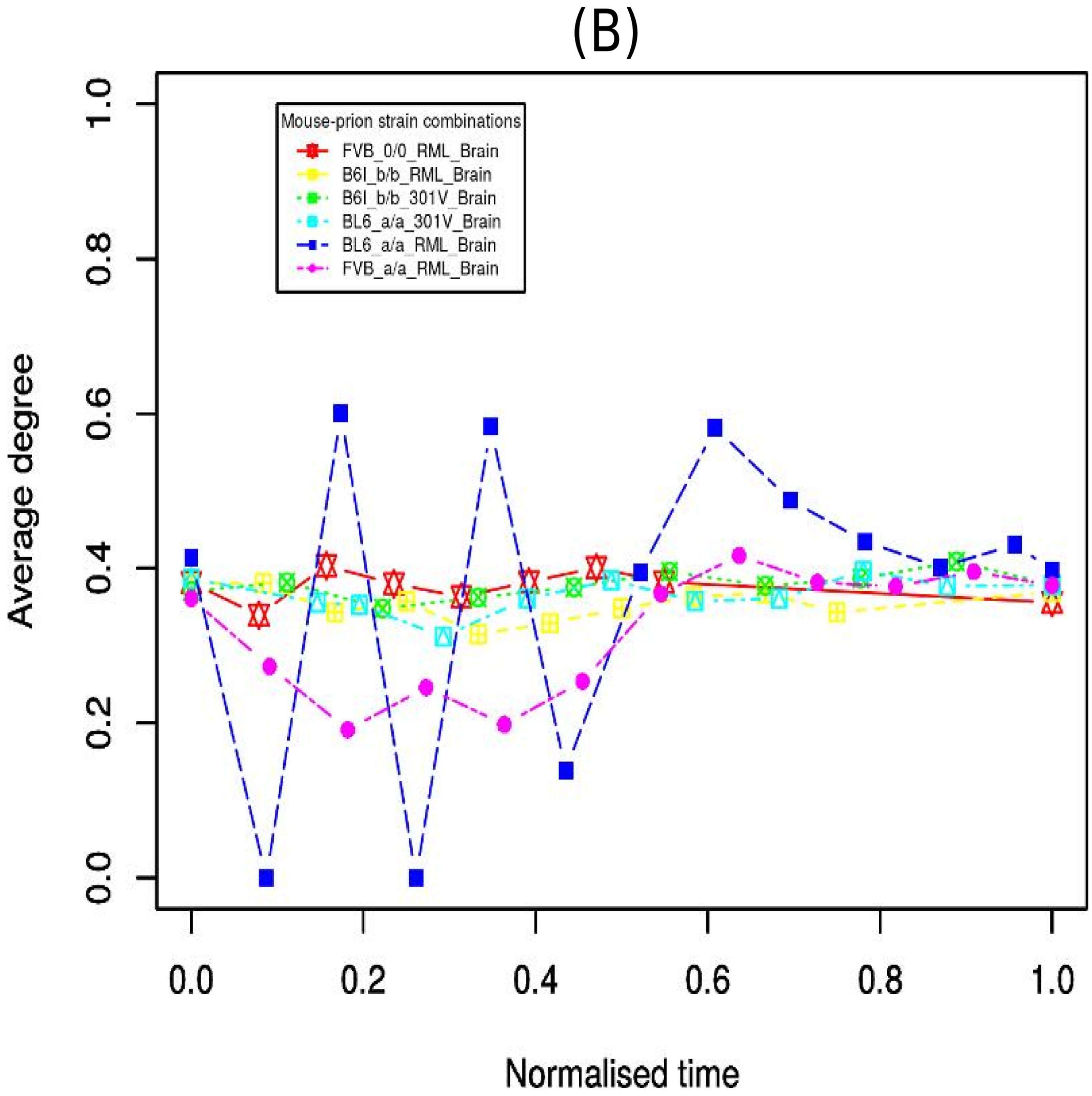


**Figure A: Temporal change in global properties of protein networks corresponding to different mouse-prion models:** For most of the mouse-prion models the average clustering coefficient does not change with time (disease progression). For the models BL6_a/a_RML_Brain and FVB_a/a_RML_Brain, the initial temporal protein networks are small (approximately less than 20 nodes). Hence, these combinations show the fluctuations in the behavior of global properties in the initial period. (A) Temporal change in the average degree of the prion disease related protein networks, corresponding to different mouse strain-prion strain combinations. (B) Temporal change in the average clustering coefficient of the prion disease related protein networks, corresponding to different mouse strain-prion strain combinations.

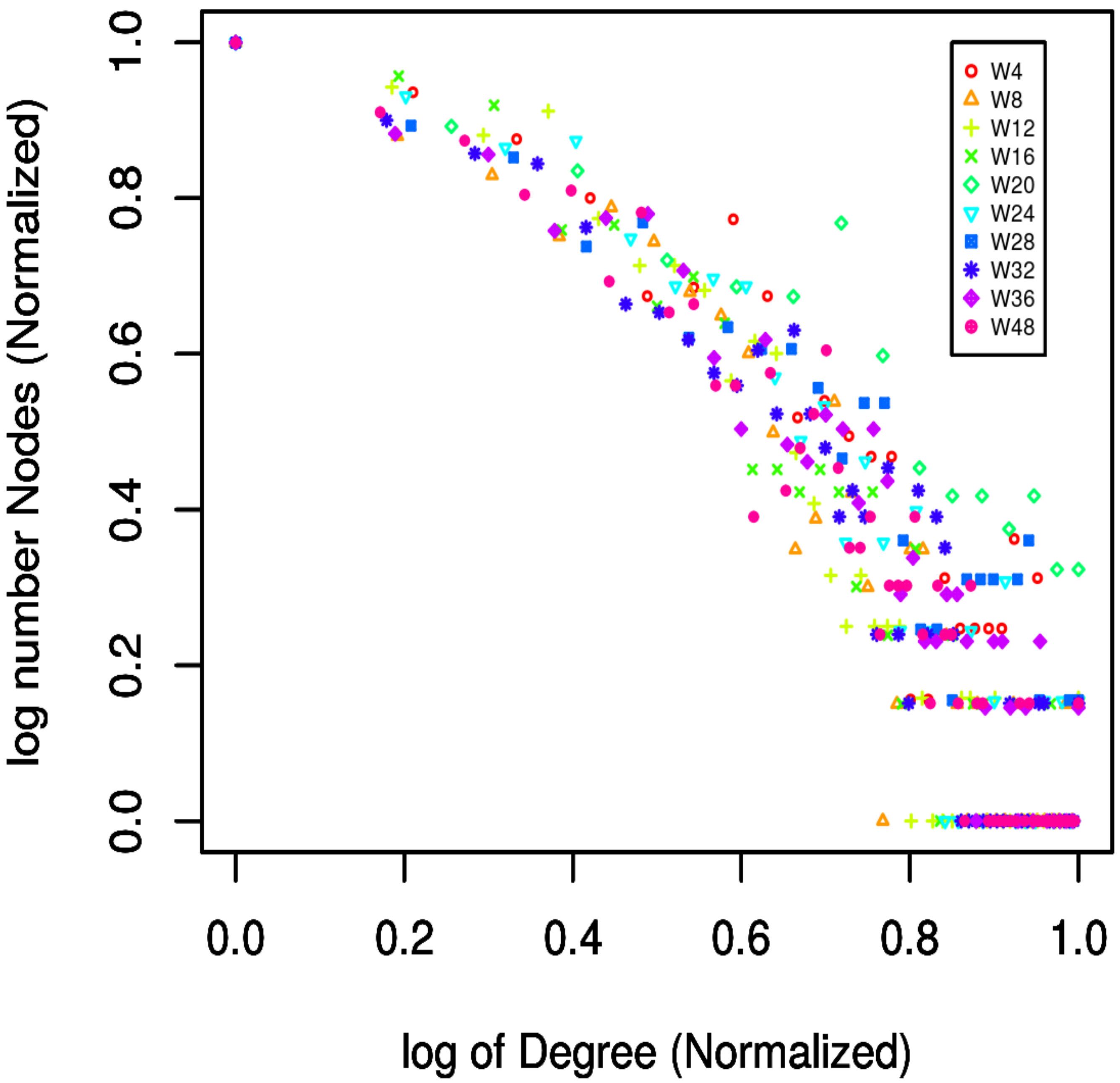


**Figure B: Degree distribution of temporal protein networks corresponding to B6.I_b/b_RML_Brain mouse-prion model:** The temporal protein networks of every mouse-prion model approximately follows the powerlaw degree distribution. The figure shows the plot for one of the mouse-prion combinations. Wi shows the plot corresponding to the week i.

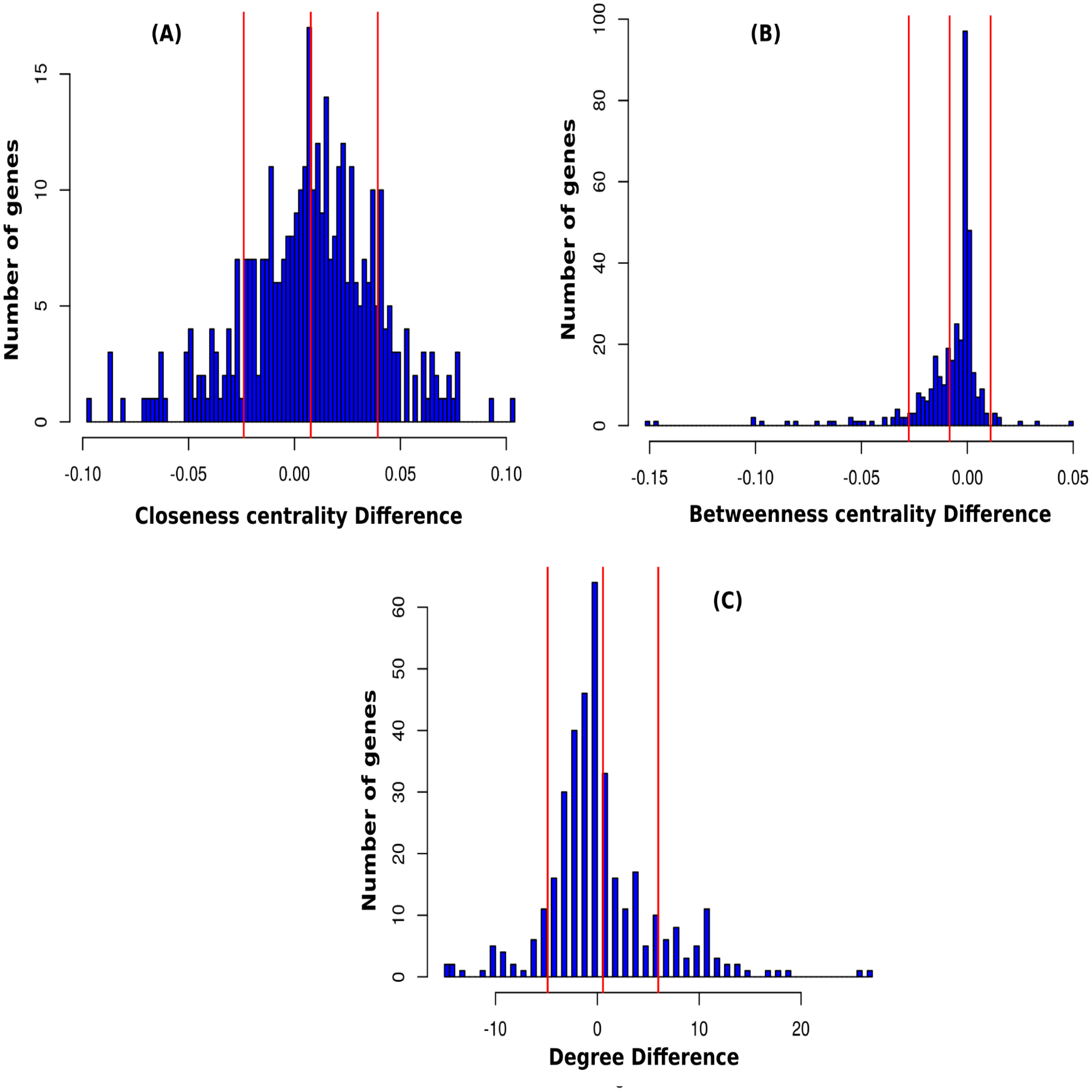


**Figure C: Histogram corresponding to centrality difference:** The left, middle and right red vertical lines in the plots shows the marks for the left standard deviation, mean and right standard deviation respectively. The genes present to the right of the right standard deviation has been taken as genes playing crucial roles in the disease progression. (A) Closeness centrality measures the closeness of a node to other nodes in the network. The graph here shows that for most of the genes in the disease network as compared to the control network, the difference in centrality is marginal. (B) Betweenness centrality measures the number of paths being traversed through a node. More paths through a node in the network make a node important from the communication point of view. This also shows the normal behavior supporting the initial hypothesis. (C) Degree measures the local communication and activity of a node in the network. The behavior of the graph is similar to other measurements.

(A)

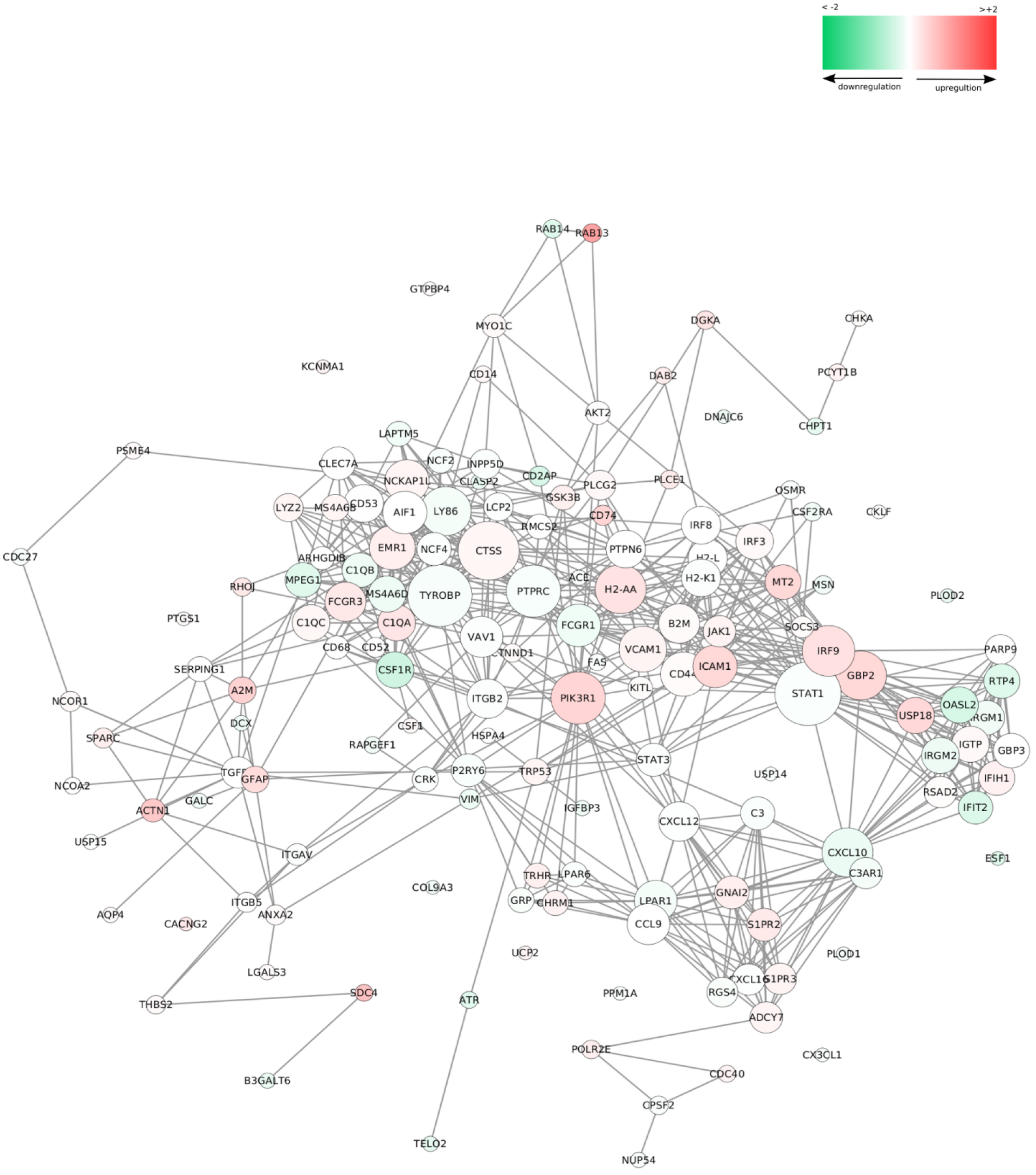
< -2
>+2
downregulation
upregultion
RAB14
RAB13
GTPBP4
MYO1C
DGKA
CHKA
KCNMA1
CD14
DAB2
PCYT1B
AKT2
DNAJC6
CHPT1
LAPTM5
PSME4
CLEC7A
NCF2
INPP5D
CD2AP
NCKAP1L
CLASP2
PLCG2
PLCE1
OSMR
LYZ2
MS4A6B
CD53
AIF1
LY86
LCP2
GSK3B
CD74
CSF2RA
CKLF
RMCS2
IRF8
EMR1
NCF4
CTSS
PTPN6
IRF3
CDC27
ARHGDIB
H2-L
C1QB
MPEG1
ACE
H2-K1
MT2
MSN
RHOJ
MS4A6D
TYROBP
PTPRC
H2-AA
PLOD2
FCGR3
PTGS1
C1QC
C1QA
FCGR1
B2M
JAK1
SOCS3
VAV1
CD68
CD52
TNND1
VCAM1
IRF9
PARP9
SERPING1
FAS
ICAM1
CD4
GBP2
RTP4
CSF1R
NCOR1
A2M
KITL
STAT1
ITGB2
PIK3R1
OASL2
USP18
RGM1
DCX
CSF1
HSPA4
IGTP
GBP3
SPARC
RAPGEF1
IRGM2
STAT3
USP14
TGF
P2RY6
TRP53
IFIH1
NCOA2
GFAP
CRK
RSAD2
VIM
GALC
ACTN1
IGFBP3
C3
IFIT2
CXCL12
USP15
CXCL10
ITGAV
ESF1
TRHR
LPAR6
C3AR1
COL9A3
GRP
CHRM1
LPAR1
ITGB5
GNAI2
AQP4
ANXA2
CACNG2
CCL9
S1PR2
UCP2
PLOD1
LGALS3
CXCL1
S1PR3
SDC4
THBS2
ATR
PPM1A
RGS4
ADCY7
POLR2E
CX3CL1
B3GALT6
CDC40
CPSF2
TELO2
NUP54

(B)

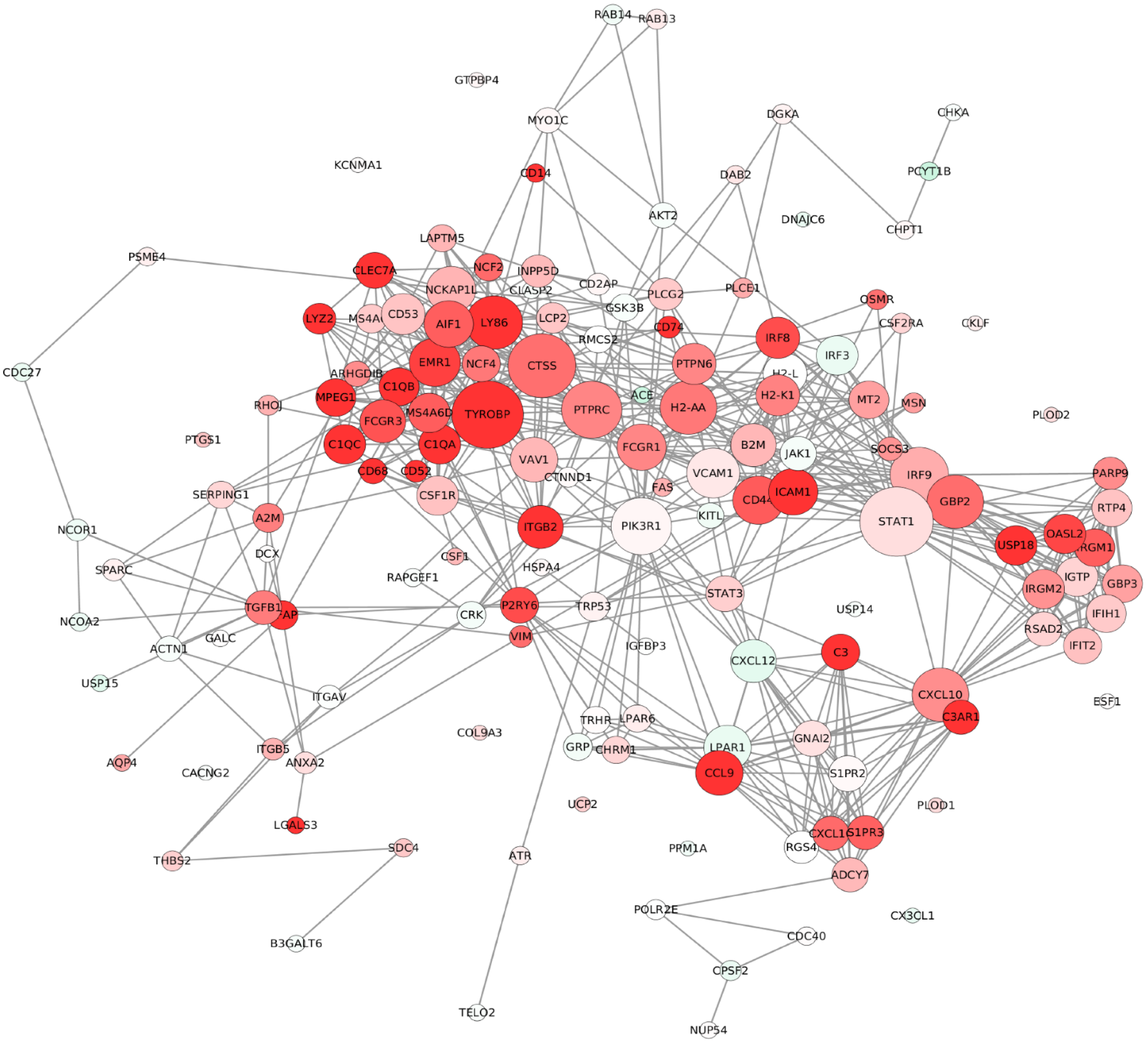


**Figure D: Networks of 148 genes with node dynamics captured as differential gene expression for B6.I-RML mouse-prion model.** All 148 genes are mapped to protein functional networks using STRING database and colors are mapped to represent up or down regulation of the corresponding genes. The present network is static network with only the node dynamics corresponding to gene differential expressions of B6.I-RML mouse-prion model. The size of the nodes/genes in the network is mapped to their respective degrees corresponding to this network. Higher degree is represented as larger node and similarly, lower degree is represented as relatively smaller sized network nodes. (A) Network at week 4. It represents the network at the initial stages of the prion disease progression. (B) Network at week 48. Represents the network at the end stages of the prion disease progression.

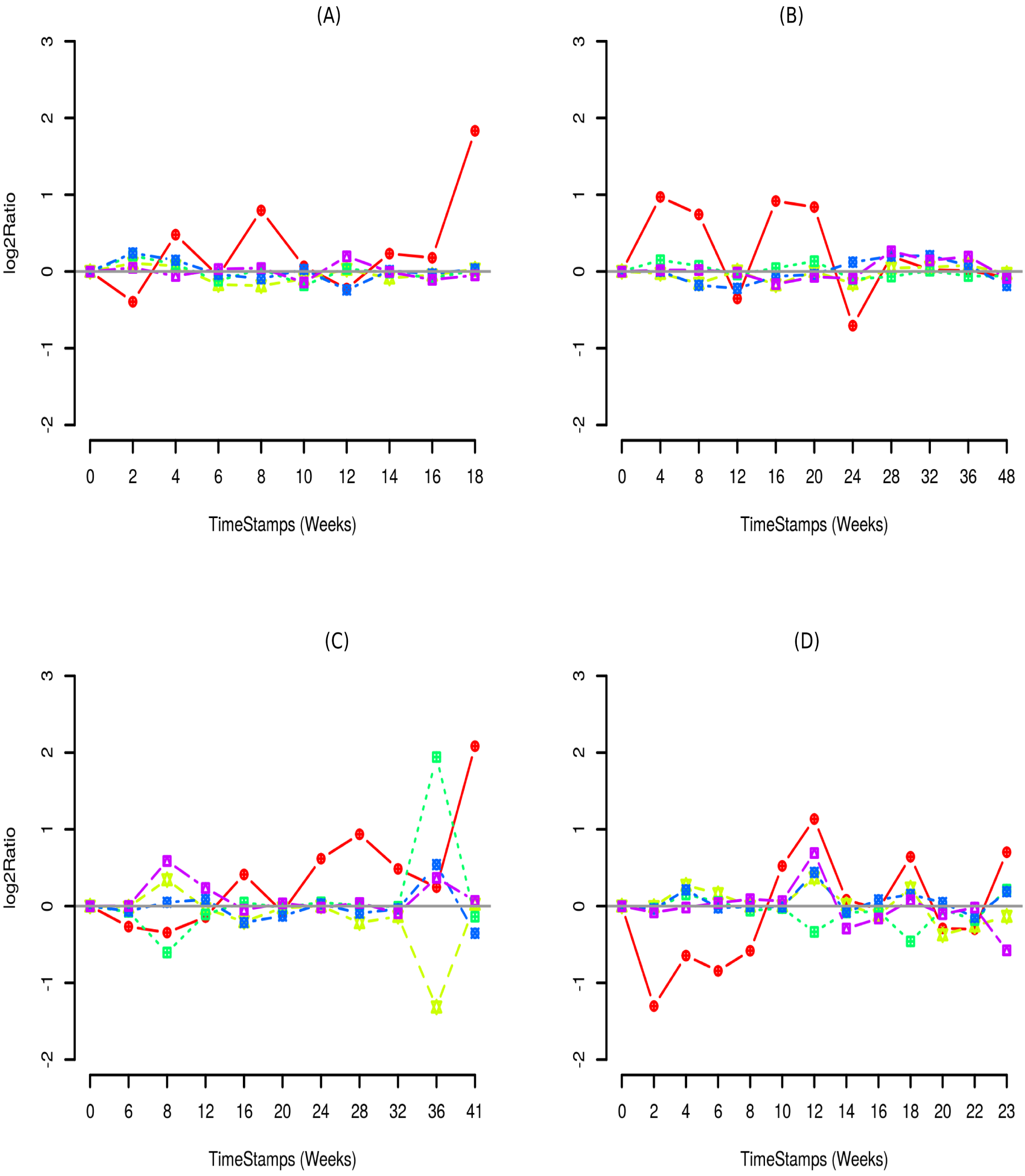
(A)
(B)
(C)
(D)
log2Ratio
TimeStamps (Weeks)

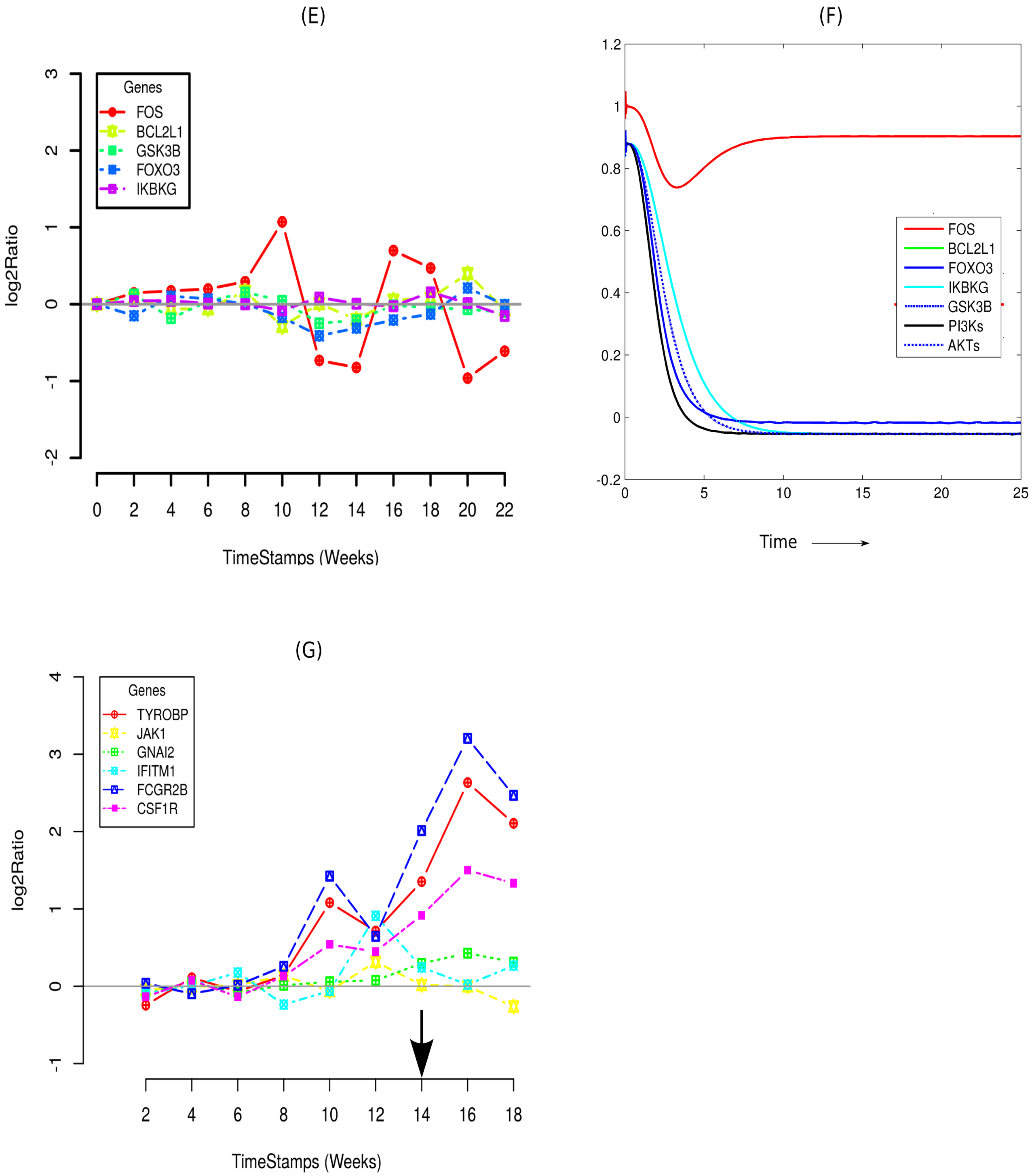


**Figure E: Comparison of microarray results with the model predictions.** Comparison of the output components (Fos, Bcl2l1, Ikbkg, Gsk3b, Foxo3) of the identified bow-tie network structure with the model prediction. Microarray results are taken from the work results of Hwang et al. [1]. (A) Differential expression results corresponding B6I-301V mouse-prion combination. (B) Differential expression results corresponding B6I-RML model. (C) Differential expression results corresponding BL6-301V model. (D) Differential expression results corresponding BL6-RML

model.(E) Differential expression results corresponding FVB-RML model. (F) Model predictions of the differential expressions of the output components. (G) Differential gene expression of the input components (Tyrobp, Ifitm1, Jak1, Fcgr2b, Gnai2, Csf1r) of the identified bow-tie signaling network structure. The presented differential expression corresponds to B6I-301V mouse-prion combination. For all other mouse-prion combinations, the differential expression pattern of these input component genes are approximately similar. The arrow on the x-axis at 14 weeks shows the time-stamp at which the input signals were used for the model predictions.

**Supplementary tables:**

**Table 1:** List of 148 shared genes

| Gene Symbols | Gene Names | Studies |
|---|---|---|
| OASL2* | 2-5 oligoadenylate synthetase-like 2 | [2] |
| CLEC7A* | C-type lectin domain family 7 member a | [3] |
| CD14* | CD14 antigen | [2] [3] |
| CD44* | CD44 antigen | [2] |
| CD52* | CD52 antigen | [4] [2] |
| CD53* | CD53 antigen | [5] [4] [2] |
| CD68* | CD68 antigen | [3] [4] [2] |
| EMR1* | EGF-like module containing mucin-like hormone receptor-like sequence 1 | [6] |
| FCGR3* | Fc receptor IgG low affinity III | [2] |
| NCKAP1L* | NCK associated protein 1 like | [3] |
| ARHGDIB* | Rho GDP dissociation inhibitor (GDI) beta | |
| TYROBP* | TYRO protein tyrosine kinase binding protein | [4] [2] |
| ADCY7* | adenylate cyclase 7 | |
| AIF1* | allograft inflammatory factor 1 | [2] |
| A2M* | alpha-2-macroglobulin | [4] [2] |
| AQP4* | aquaporin 4 | [4] |
| B2M* | beta-2 microglobulin | [7] [8] [5] [4] [2] [6] |
| CTSS* | cathepsin S | [7] [8] [5] [4] [2] [3] [6] |
| CCL9* | chemokine (C-C motif) ligand 9 | [3] |
| CXCL10* | chemokine (C-X-C motif) ligand 10 | [2] |
| CSF1* | colony stimulating factor 1 (macrophage) | |
| CSF1R* | colony stimulating factor 1 receptor | [8] [5] [4] |
| C1QC* | complement component 1 q subcomponent C chain | [3] [2] |
| C1QA* | complement component 1 q subcomponent alpha polypeptide | [3] [2] [8] [4] |
| C1QB* | complement component 1 q subcomponent beta polypeptide | [3] [2] [8] [4] [6] |
| C3* | complement component 3 | [4] |
| C3AR1* | complement component 3a receptor 1 | [3] [2] [4] |
| GFAP* | glial fibrillary acidic protein | [3] [2] [7] [8] [4] |
| GBP2* | guanylate binding protein 2 | [2] |
| H2-D1* | histocompatibility 2 D region | [2] [8] |
| H2-K1* | histocompatibility 2 K1 K region | [2] |
| H2-AA* | histocompatibility 2 class II antigen A alpha | |
| H2-AB1* | histocompatibility 2 class II antigen A beta 1 | |
| INPP5D* | inositol polyphosphate-5-phosphatase D | [3] |
| ITGB2* | integrin beta 2 | [3] [2] [4] |
| ITGB5* | integrin beta 5 | |
| IGTP* | interferon gamma induced GTPase | |
| IFIH1* | interferon induced with helicase C domain 1 | |
| IRF8* | interferon regulatory factor 8 | [3] |

Continued on next page

**Table 1 – continued from previous page**

| Gene Symbols | Gene Names | Studies |
|---|---|---|
| IFIT2* | interferon-induced protein with tetratricopeptide repeats 2 | |
| LGALS3* | lectin galactose binding soluble 3 | [2] [4] |
| LAPTM5* | lysosomal-associated protein transmembrane 5 | [7] [5] [2] |
| MPEG1* | macrophage expressed gene 1 | [3] [2] |
| MS4A6B* | membrane-spanning 4-domains subfamily A member 6B | [4] |
| MS4A6D* | membrane-spanning 4-domains subfamily A member 6D | [2] |
| MT2* | metallothionein 2 | [6] |
| MSN* | moesin | [4] [2] |
| OSMR* | oncostatin M receptor | [3] [4] |
| PLCE1* | phospholipase C epsilon 1 | |
| PTGS1* | prostaglandin-endoperoxide synthase 1 | |
| PTPN6* | protein tyrosine phosphatase non-receptor type 6 | |
| PTPRC* | protein tyrosine phosphatase receptor type C | [2] |
| P2RY6* | pyrimidinergic receptor P2Y G-protein coupled 6 | |
| RHOJ* | ras homolog gene family member J | [3] |
| SERPING1* | serine (or cysteine) peptidase inhibitor clade G member 1 | [8] |
| STAT1* | signal transducer and activator of transcription 1 | |
| STAT3* | similar to Stat3- signal transducer and activator of transcription 3 | [3] [8] |
| SOCS3* | suppressor of cytokine signaling 3 | [3] [2] |
| SDC4* | syndecan 4 | [8] |
| THBS2* | thrombospondin 2 | |
| TGFB1* | transforming growth factor beta 1 | [7] |
| UCP2* | uncoupling protein 2 (mitochondrial proton carrier) | [3] |
| VIM* | vimentin | [8] [2] |
| CD2AP | CD2-associated protein | |
| CD74 | CD74 antigen (invariant polypeptide of major histocompatibility complex class II antigen-associated) | |
| CLASP2 | CLIP associating protein 2 | |
| DNAJC6 | DnaJ (Hsp40) homolog subfamily C member 6 | |
| ESF1 | ESF1 nucleolar pre-rRNA processing protein homolog (S. cerevisiae) | |
| FAS | Fas (TNF receptor superfamily member 6) | |
| FCGR1 | Fc receptor IgG high affinity I | [2] |
| GTPBP4 | GTP binding protein 4 | [7] |
| JAK1 | Janus kinase 1 | |
| RAB13 | RAB13 member RAS oncogene family | |
| RAB14 | RAB14 member RAS oncogene family | |
| CHPT1 | RIKEN cDNA 7120451J01 gene- choline phosphotransferase 1 | |
| RAPGEF1 | Rap guanine nucleotide exchange factor (GEF) 1 | |
| TELO2 | TEL2 telomere maintenance 2 homolog (S. cerevisiae) | |
| ACTN1 | actinin alpha 1 | |
| ACE | angiotensin I converting enzyme (peptidyl-dipeptidase A) 1 | |
| ATR | ataxia telangiectasia and Rad3 related | |
| CACNG2 | calcium channel voltage-dependent gamma subunit 2 | |
| CTNND1 | catenin (cadherin associated protein) delta 1 | |

Continued on next page

**Table 1 – continued from previous page**

| Gene Symbols | Gene Names | Studies |
|---|---|---|
| CDC27 | cell division cycle 27 homolog (S. cerevisiae) | |
| CDC40 | cell division cycle 40 homolog (yeast) | |
| CXCL12 | chemokine (C-X-C motif) ligand 12 | [2] |
| CXCL16 | chemokine (C-X-C motif) ligand 16 | [2] |
| CX3CL1 | chemokine (C-X3-C motif) ligand 1 | |
| CKLF | chemokine-like factor | |
| CHKA | choline kinase alpha | |
| CHRM1 | cholinergic receptor muscarinic 1 CNS | |
| CPSF2 | cleavage and polyadenylation specific factor 2 | |
| COL9A3 | collagen type IX alpha 3 | |
| CSF2RA | colony stimulating factor 2 receptor alpha low-affinity (granulocyte-macrophage) | |
| DGKA | diacylglycerol kinase alpha | |
| DAB2 | disabled homolog 2 (Drosophila) | [2] [5] |
| DCX | doublecortin | |
| GALC | galactosylceramidase | |
| GRP | actinin alpha 1 | |
| GSK3B | glycogen synthase kinase 3 beta | |
| GNAI2 | guanine nucleotide binding protein (G protein) alpha inhibiting 2 | |
| GBP3 | guanylate binding protein 3 | |
| HSPA4 | heat shock protein 4 | |
| IRGM1 | immunity-related GTPase family M member 1 | |
| IRGM2 | immunity-related GTPase family M member 2 | |
| IGFBP3 | insulin-like growth factor binding protein 3 | |
| ITGAV | integrin alpha V | |
| ICAM1 | intercellular adhesion molecule 1 | [2] |
| IRF3 | interferon regulatory factor 3 | |
| IRF9 | interferon regulatory factor 9 | |
| KITL | kit ligand | |
| LY86 | lymphocyte antigen 86 | [5] [7] [2] |
| LCP2 | lymphocyte cytosolic protein 2 | |
| LPAR1 | lysophosphatidic acid receptor 1 | |
| LYZ2 | lysozyme 2 | [2] |
| NCF2 | neutrophil cytosolic factor 2- neutrophil cytosolic factor 2 related sequence | |
| NCF4 | neutrophil cytosolic factor 4 | |
| NCOR1 | nuclear receptor co-repressor 1 | |
| NCOA2 | nuclear receptor coactivator 2 | |
| NUP54 | nucleoporin 54 | |
| PCYT1B | phosphate cytidylyltransferase 1 choline beta isoform | |
| PIK3R1 | phosphatidylinositol 3-kinase regulatory subunit polypeptide 1 (p85 alpha) | |
| PLCG2 | phospholipase C gamma 2 | |
| PARP9 | poly (ADP-ribose) polymerase family member 9 | |
| POLR2E | polymerase (RNA) II (DNA directed) polypeptide E | |

Continued on next page

**Table 1 – continued from previous page**

| Gene Symbols | Gene Names | Studies |
|---|---|---|
| KCNMA1 | potassium large conductance calcium-activated channel subfamily M alpha member 1 | |
| PLOD2 | procollagen lysine 2-oxoglutarate 5-dioxygenase 2 | |
| PLOD1 | procollagen-lysine 2-oxoglutarate 5-dioxygenase 1 | |
| PSME4 | proteasome (prosome macropain) activator subunit 4 | |
| PPM1A | protein phosphatase 1A magnesium dependent alpha isoform | |
| LPAR6 | purinergic receptor P2Y G-protein coupled 5 | |
| RSAD2 | radical S-adenosyl methionine domain containing 2 | |
| RTP4 | receptor transporter protein 4 | |
| RGS4 | regulator of G-protein signaling 4 | |
| SPARC | secreted acidic cysteine rich glycoprotein- similar to Secreted acidic cysteine rich glycoprotein | [5] |
| ANXA2 | annexin A2 | |
| AKT2 | Protein kinase Akt-2 | |
| B3GALT6 | UDP-Gal:betaGal beta 1 3-galactosyltransferase polypeptide 6 | |
| MYO1C | similar to nuclear myosin I beta- myosin IC | |
| S1PR2 | sphingosine-1-phosphate receptor 2 | |
| S1PR3 | sphingosine-1-phosphate receptor 3 | |
| TRHR | thyrotropin releasing hormone receptor | |
| TRP53 | transformation related protein 53 | |
| USP14 | ubiquitin specific peptidase 14 | |
| USP15 | ubiquitin specific peptidase 15 | |
| USP18 | ubiquitin specific peptidase 18- similar to ubiquitin specific protease UBP43 | |
| CRK | v-crk sarcoma virus CT10 oncogene homolog (avian) | |
| VCAM1 | vascular cell adhesion molecule 1 | |
| VAV1 | vav 1 oncogene | |

(a)Genes with asterisks are common DEGs found in both this work and Hwang's work.

**Table 2:** List of pathways with corresponding genes from 148 shared DEGs

| KEGG Pathway Names | Gene Symbols | Count |
|---|---|---|
| Cell adhesion molecules (CAMs)* | Ptprc Sdc4 Vcam1 H2-D1 H2-K1 H2-AB1 H2-AA Itgav Itgb2 Icam1 | 10 |
| Antigen processing and presentation* | H2-D1 H2-K1 H2-AB1 H2-AA Hspa4 Ctss B2m Cd74 | 8 |
| Hematopoietic cell lineage* | Cd14 Cd44 Fcgr1 Csf1 Csf1r Csf2ra H2-AA Kitl | 8 |
| Phosphatidylinositol signaling system | Dgka Inpp5d Pik3r1 Plce1 Plcg2 | 5 |
| Natural killer cell mediated cytotoxicity* | Fas Fcgr3 Tyrobp H2-D1 H2-K1 Itgb2 Icam1 Lcp2 Pik3r1 Plcg2 Ptpn6 Vav1 | 12 |
| Leukocyte transendothelial migration* | Actn1 Ctnnd1 Cxcl10 Gnai2 Itgb2 Icam1 Msn Ncf2 Ncf4 Pik3r1 Plcg2 Vcam1 Vav1 | 13 |
| Neurotrophin signaling pathway* | Rapgef1 Arhgdib Gsk3b Pik3r1 Plcg2 Akt2 Trp53 Crk | 8 |
| Chemokine signaling pathway* | Adcy7 Ccl9 Cxcl10 Cxcl12 Cxcl16 Cx3cl1 Gsk3b Gnai2 Pik3r1 Stat1 Akt2 Stat3 Crk Vav1 | 14 |
| Fc gamma R-mediated phagocytosis* | Fcgr1 Inpp5d Pik3r1 Plcg2 Ptprc Akt2 Crk Vav1 | 8 |
| Regulation of actin cytoskeleton* | Cd14 Nckap1l Actn1 Chrm1 Itgav Itgb2 Itgb5 Msn Pik3r1 Crk Vav1 | 11 |
| Insulin signaling pathway | Rapgef1 Gsk3b Inpp5d Pik3r1 Akt2 Socs3 Crk | 7 |
| Cytokine-cytokine receptor interaction* | Fas Ccl9 Cxcl10 Cxcl12 Cxcl16 Cx3cl1 Csf1 Tgfb1 Osmr Kitl Csf2ra Csf1r | 12 |
| Jak-STAT signaling pathway* | Jak1 Csf2ra Irf9 Osmr Pik3r1 Ptpn6 Stat1 Akt2 Stat3 Socs3 | 10 |
| Complement and coagulation cascades* | A2m C1qa C1qb C1qc C3 C3ar1 Serping1 | 7 |
| Endocytosis | Dnajc6 Csf1r Dab2 H2-D1 H2-K1 | 5 |
| Lysosome | Cd68 Ctss Galc Laptm5 | 4 |
| Glycerophospholipid metabolism | Chpt1 Chka Pcyt1b | 3 |
| Focal adhesion* | Thbs2 Rapgef1 Crk Vav1 Actn1 Itgav Itgb5 Pik3r1 Akt2 | 9 |
| Calcium signaling pathway | Adcy7 Chrm1 Plce1 Plcg2 Trhr | 5 |
| Toll-like receptor signaling pathway* | Irf3 Cd14 Cxcl10 Pik3r1 Stat1 Akt2 | 6 |
| MAPK signaling pathway | Cd14 Fas Cacng2 Ppm1a Akt2 Trp53 Tgfb1 Crk | 8 |
| Neuroactive ligand-receptor interaction | Chrm1 C3ar1 Lpar1 Lpar6 P2ry6 S1pr2 S1pr3 Trhr | 8 |
| Tight junction | Rab13 Actn1 Gnai2 Akt2 | 4 |
| B cell receptor signaling pathway* | Gsk3b Inpp5d Pik3r1 Plcg2 Ptpn6 Akt2 Vav1 | 7 |
| T cell receptor signaling pathway* | Lcp2 Gsk3b Pik3r1 Ptpn6 Ptprc Akt2 Vav1 | 7 |

Continued on next page

**Table 2 – continued from previous page**

| KEGG Pathway Names | Gene Symbols | Count |
|---|---|---|
| Lysine degradation | Plod1 Plod2 | 2 |
| Renin-angiotensin system | Ace | 1 |
| p53 signaling pathway | Atr Fas Igfbp3 Trp53 | 4 |
| Ubiquitin mediated proteolysis | Cdc27 Socs3 | 2 |
| RI-I-like receptor signaling pathway | Cxcl10 Ifih1 Irf3 | 3 |
| Spliceosome | Cdc40 | 1 |
| Purine metabolism | Polr2e | 1 |
| Arachidonic acid metabolism | Ptgs1 | 1 |
| Proteosome | Psme4 | 1 |
| Vascular smooth muscle contraction | Kcnma1 | 1 |
| Heparan sulfate biosynthesis | B3galt6 | 1 |

(a) The pathways marked with asterisks are found to be enriched (P value $< 0.05$) in the set of 148 shared DEGs.

**Table 3:** Network information for B6.I-Prnp(b/b)-301V combination:

| **Dataset type** | **W2*** | **W4** | **W6** | **W8** | **W10** | **W12** | **W14** | **W16** | **W18** |
|---|---|---|---|---|---|---|---|---|---|
| Number of DEGs | 1590 | 1557 | 1664 | 1609 | 1690 | 1655 | 1715 | 1775 | 1779 |
| Number of mapped proteins | 539 | 465 | 539 | 475 | 599 | 520 | 620 | 614 | 699 |
| Number of nodes in LCC | 415 | 386 | 432 | 421 | 498 | 435 | 526 | 516 | 588 |
| Number of edges in LCC | 1119 | 1078 | 1104 | 1001 | 1497 | 1188 | 1732 | 1839 | 2124 |

Mouse strain = C57BL/6I-1, Prion strain = 301V mouse adapted BSE prions, Host genotype = b/b

For filtering the DEGs at a particular time-stamp, we have used a p-value threshold ($< 0.05$). Same criteria has been used for the DEGs identification across different mouse strain-prion strain combinations.

* Wi represents the network at week i

**Table 4:** Network information for B6.I-Prnp(b/b)-RML combination:

| **Dataset type** | **W4** | **W8** | **W12** | **W16** | **W20** | **W24** | **W28** | **W32** | **W36** | **W48** |
|---|---|---|---|---|---|---|---|---|---|---|
| Number of DEGs | 1545 | 1575 | 1584 | 1658 | 1636 | 1649 | 1673 | 1697 | 1689 | 1654 |
| Number of mapped proteins | 461 | 506 | 500 | 537 | 393 | 498 | 479 | 572 | 589 | 615 |
| Number of nodes in LCC | 379 | 412 | 404 | 433 | 277 | 407 | 364 | 478 | 517 | 527 |
| Number of edges in LCC | 960 | 1069 | 1023 | 1014 | 590 | 1004 | 999 | 1625 | 1570 | 1988 |

Mouse strain = C57BL/6I-1, Prion strain = Rocky mountain lab (RML) mouse adapted scrapie prions, Host genotype = b/b

**Table 5:** Network information for BL6-Prnp(a/a)-301V combination:

| **Dataset type** | **W6** | **W8** | **W12** | **W16** | **W20** | **W24** | **W28** | **W32** | **W36** | **W48** |
|---|---|---|---|---|---|---|---|---|---|---|
| Number of DEGs | 1738 | 1767 | 1610 | 1593 | 1655 | 1715 | 1729 | 1571 | 1721 | 1668 |
| Number of mapped proteins | 556 | 580 | 446 | 522 | 509 | 585 | 619 | 449 | 447 | 546 |
| Number of nodes in LCC | 467 | 480 | 389 | 421 | 426 | 495 | 541 | 378 | 324 | 438 |
| Number of edges in LCC | 1164 | 1323 | 964 | 976 | 1076 | 1528 | 1649 | 1199 | 717 | 1411 |

Mouse strain = C57BL/6J, Prion strain = 301V mouse adapted BSE prions, Host genotype = a/a

**Table 6:** Network information for BL6-Prnp(a/a)-RML combination:

| **Data-type** | **W2** | **W4** | **W6** | **W8** | **W10** | **W12** | **W14** | **W16** | **W18** | **W20** | **W22** | **W23** |
|---|---|---|---|---|---|---|---|---|---|---|---|---|
| DEGs | 135 | 41 | 49 | 48 | 36 | 4085 | 122 | 241 | 1760 | 897 | 648 | 1981 |
| prote-ins* | 17 | 5 | 9 | 4 | 12 | 1035 | 61 | 109 | 496 | 339 | 241 | 700 |
| nodes | 7 | 3 | 3 | 4 | 7 | 925 | 59 | 105 | 431 | 290 | 216 | 641 |
| edges | 6 | 3 | 2 | 4 | 9 | 2962 | 250 | 382 | 1292 | 1007 | 834 | 2112 |

Mouse strain = C57BL/6J, Prion strain = Rocky mountain lab (RML) mouse adapted scrapie prions, Host genotype =a/a

*The small number of nodes in the early time-stamped networks is the cumulative effect of both incomplete knowledge and less number of DEGs at these time-points.

**Table 7:** Network information for FVB-Prnp(a/a)-RML combination:

| Dataset type | W2 | W4 | W6 | W8 | W10 | W12 | W14 | W16 | W18 | W20 | W22 |
|---|---|---|---|---|---|---|---|---|---|---|---|
| DEGs | 582 | 657 | 586 | 466 | 635 | 1194 | 1077 | 935 | 950 | 3734 | 1852 |
| proteins | 135 | 166 | 156 | 84 | 165 | 407 | 397 | 339 | 355 | 1283 | 666 |
| nodes | 91 | 73 | 30 | 46 | 117 | 316 | 322 | 288 | 287 | 1191 | 587 |
| edges | 124 | 117 | 36 | 66 | 208 | 861 | 854 | 796 | 871 | 4311 | 2101 |

Mouse strain = FVB/NCr, Prion strain = Rocky mountain lab (RML) mouse adapted scrapie prions, Host genotype = a/a

**Table 8:** Network information for FVB-Prnp(0/0)-RML combination:

| Dataset type | W4 | W8 | W12 | W16 | W20 | W24 | W28 | W51 |
|---|---|---|---|---|---|---|---|---|
| Number of DEGs | 1659 | 1714 | 1714 | 1754 | 1699 | 1823 | 1630 | 1595 |
| Number of mapped proteins | 514 | 556 | 565 | 552 | 527 | 599 | 510 | 540 |
| Number of nodes in LCC | 442 | 472 | 452 | 420 | 443 | 501 | 400 | 461 |
| Number of edges in LCC | 1168 | 1153 | 1203 | 1051 | 1073 | 1360 | 1009 | 1185 |

Mouse strain = FVB.129-prnp, Prion strain = Rocky mountain lab (RML) mouse adapted scrapie prions, Host genotype = 0/0

**Table 9:** Global properties details for B6.I-Prnp(b/b)-301V combination:

| Property | W2* | W4 | W6 | W8 | W10 | W12 | W14 | W16 | W18 |
|---|---|---|---|---|---|---|---|---|---|
| Avg. clustering coeff. | 0.38 | 0.35 | 0.36 | 0.38 | 0.40 | 0.38 | 0.39 | 0.41 | 0.38 |
| Centralization | 0.08 | 0.09 | 0.07 | 0.07 | 0.09 | 0.08 | 0.09 | 0.11 | 0.09 |
| Avg. shortest path | 4.56 | 5.00 | 5.31 | 4.99 | 4.61 | 4.35 | 4.99 | 4.22 | 4.28 |
| Density | 0.01 | 0.01 | 0.01 | 0.01 | 0.01 | 0.01 | 0.01 | 0.01 | 0.01 |
| Network heterogeneity | 0.99 | 0.99 | 1.05 | 0.95 | 1.05 | 1.03 | 1.02 | 1.07 | 1.06 |
| Power-law coefficient | 1.51 | 1.48 | 1.48 | 1.48 | 1.61 | 1.51 | 1.48 | 1.41 | 1.45 |

**Table 10:** Global properties details for B6.I-Prnp(b/b)-RML combination:

| Property | W4 | W8 | W12 | W16 | W20 | W24 | W28 | W32 | W36 | W48 |
|---|---|---|---|---|---|---|---|---|---|---|
| Avg. clustering coeff. | 0.38 | 0.34 | 0.36 | 0.31 | 0.33 | 0.35 | 0.36 | 0.37 | 0.34 | 0.37 |
| Centralization | 0.06 | 0.08 | 0.09 | 0.07 | 0.04 | 0.06 | 0.06 | 0.08 | 0.06 | 0.09 |
| Avg. shortest path | 4.65 | 4.53 | 4.80 | 5.03 | 5.48 | 4.76 | 4.62 | 4.36 | 4.79 | 4.26 |
| Density | 0.01 | 0.01 | 0.01 | 0.01 | 0.02 | 0.01 | 0.02 | 0.01 | 0.01 | 0.01 |
| Network heterogeneity | 0.93 | 0.98 | 0.93 | 1.01 | 0.81 | 0.94 | 0.97 | 1.02 | 1.01 | 1.05 |
| Power-law coefficient | 1.47 | 1.53 | 1.54 | 1.58 | 1.44 | 1.57 | 1.37 | 1.45 | 1.48 | 1.40 |

**Table 11:** Global properties details for BL6-Prnp(a/a)-301V combination

| Property | W6 | W8 | W12 | W16 | W20 | W24 | W28 | W32 | W36 | W48 |
|---|---|---|---|---|---|---|---|---|---|---|
| Avg. clustering coeff. | 0.36 | 0.35 | 0.31 | 0.36 | 0.38 | 0.36 | 0.36 | 0.40 | 0.38 | 0.38 |
| centralization | 0.06 | 0.06 | 0.07 | 0.07 | 0.06 | 0.07 | 0.06 | 0.10 | 0.08 | 0.10 |
| Avg. shortest path | 4.69 | 4.63 | 4.88 | 5.54 | 4.97 | 4.14 | 4.65 | 4.56 | 5.54 | 4.46 |
| density | 0.01 | 0.01 | 0.01 | 0.01 | 0.01 | 0.01 | 0.01 | 0.02 | 0.01 | 0.01 |
| Network heterogeneity | 0.95 | 0.97 | 1.00 | 0.89 | 0.94 | 1.03 | 0.95 | 1.04 | 0.95 | 0.95 |
| Power-law coefficient | 1.63 | 1.53 | 1.52 | 1.66 | 1.55 | 1.46 | 1.57 | 1.39 | 1.55 | 1.46 |

**Table 12:** Global properties details for BL6-Prnp(a/a)-RML combination

| Property | W12 | W14 | W16 | W18 | W20 | W22 | W23 |
|---|---|---|---|---|---|---|---|
| Avg. clustering coeff. | 0.40 | 0.58 | 0.49 | 0.43 | 0.40 | 0.43 | 0.40 |
| Centralization | 0.05 | 0.30 | 0.26 | 0.08 | 0.14 | 0.18 | 0.06 |
| Avg. shortest path | 4.96 | 2.62 | 3.36 | 4.84 | 3.99 | 3.48 | 4.55 |
| Density | 0.01 | 0.14 | 0.07 | 0.01 | 0.02 | 0.04 | 0.01 |
| Network heterogeneity | 1.05 | 0.68 | 0.89 | 1.02 | 1.02 | 0.98 | 1.00 |
| Power-law coefficient | 1.63 | 1.01 | 1.09 | 1.45 | 1.26 | 1.13 | 1.54 |

For the time-points earlier than 12 weeks, the number of DEGs were very less resulting in sparse disconnected network.

Calculating the global properties for these networks didn't make sense.

**Table 13:** Global properties details for FVB-Prnp(a/a)-RML combination

| **Property** | **W12** | **W14** | **W16** | **W18** | **W20** | **W22** |
|---|---|---|---|---|---|---|
| Avg. clustering coeff. | 0.37 | 0.42 | 0.38 | 0.38 | 0.40 | 0.38 |
| Centralization | 0.08 | 0.10 | 0.09 | 0.10 | 0.05 | 0.08 |
| Avg. shortest path | 4.84 | 4.87 | 4.52 | 4.13 | 4.64 | 4.27 |
| Density | 0.02 | 0.02 | 0.02 | 0.02 | 0.01 | 0.01 |
| Network heterogeneity | 0.93 | 0.97 | 0.98 | 0.94 | 1.08 | 1.10 |
| Power-law coefficient | 1.41 | 1.43 | 1.36 | 1.34 | 1.66 | 1.41 |

For the time-points earlier than 12 weeks, the number of DEGs were very less resulting in sparse disconnected network.

**Table 14:** Global properties details for FVB-Prnp(0/0)-RML combination

| **Property** | **W4** | **W8** | **W12** | **W16** | **W20** | **W24** | **W28** | **W51** |
|---|---|---|---|---|---|---|---|---|
| Avg. clustering coeff. | 0.34 | 0.40 | 0.38 | 0.36 | 0.38 | 0.40 | 0.38 | 0.36 |
| centralization | 0.08 | 0.06 | 0.06 | 0.07 | 0.06 | 0.06 | 0.07 | 0.09 |
| Avg. shortest path | 4.65 | 4.88 | 4.92 | 4.61 | 5.01 | 4.91 | 4.68 | 4.78 |
| density | 0.01 | 0.01 | 0.01 | 0.01 | 0.01 | 0.01 | 0.01 | 0.01 |
| Network heterogeneity | 1.03 | 0.88 | 0.91 | 0.99 | 0.93 | 0.90 | 0.95 | 1.02 |
| Power-law coefficient | 1.53 | 1.66 | 1.59 | 1.54 | 1.62 | 1.63 | 1.54 | 1.52 |

## References


[1] Hwang D, Lee I, Yoo H, Gehlenborg N, Cho J, Petritis B, et al. A systems approach to prion disease. Molecular systems biology. 2009;5:252.

[2] Xiang W, Windl O, Wünsch G, Dugas M, Kohlmann A, Dierkes N, et al. Identification of differentially expressed genes in scrapie-infected mouse brains by using global gene expression technology. Journal of Virology. 2004;78(20):11051–11060.

[3] Xiang W, Hummel M, Mitteregger G, Pace1 C, Windl O, Mansmann U, et al. Transcriptome analysis reveals altered cholesterol metabolism during the neurodegeneration in mouse scrapie model. Journal of Neurochemistry. 2007;102(3):834–847.

[4] Riemer C, Neidhold S, Burwinkel M, Schwarz A, Schultz J, Krätzschmar J, et al. Gene expression profiling of scrapie-infected brain tissue. Biochemical and biophysical research communications. 2004;323(2):556–564.

[5] Booth S, Bowman C, Baumgartner R, Sorensen G, Robertson C, Coulthart M, et al. Identification of central nervous system genes involved in the host response to the scrapie agent during preclinical and clinical infection. The Journal of General Virology. 2004;85(11):3459–3471.

[6] Dandoy-Dron F, Guillo F, Benboudjema L, Deslys J, Lasmézas C, Dormont D, et al. Gene expression in scrapie. cloning of a new scrapie-responsive gene and the identification of increased levels of seven other mrna transcripts. The Journal of Biological Chemistry. 1998;273(13):7691–7697.

[7] Sorensen G, Medina S, Parchaliuk D, Phillipson C, Robertson C, Booth SA. Comprehensive transcriptional profiling of prion infection in mouse models reveals networks of responsive genes. BMC Genomics. 2008;doi:10.1186/1471–2164–9–114.

[8] Skinner PJ, Abbassi1 H, Chesebro B, Race RE, Reilly C, Haase AT. Gene expression alterations in brains of mice infected with three strains of scrapie. BMC Genomics. 2006;doi:10.1186/1471–2164–7–114.
